# Supplementary material for: Organic fertilization co-selects genetically linked antibiotic and metal(loid) resistance genes in global soil microbiome
Source: Nat Commun. 2024 Jun 17;15:5168. doi: 10.1038/s41467-024-49165-5 (PMC11183072; doi:10.1038/s41467-024-49165-5)
Supplement: Supplementary file 1 — Supplementary Information [file 41467_2024_49165_MOESM1_ESM.pdf]

Supplementary Information

Organic fertilization co-selects genetically linked antibiotic and metal(loid) resistance genes in global soil microbiome

Zi-Teng Liu[1], Rui-Ao Ma[1], Dong Zhu[2], Konstantinos T. Konstantinidis[3], Yong-Guan Zhu[4,5], and Si-Yu Zhang[1*]

[1] Shanghai Key Lab for Urban Ecological Processes and Eco-Restoration, School of Ecological and Environmental Sciences, East China Normal University, Shanghai, China.

[2] Key Laboratory of Urban Environment and Health, Ningbo Observation and Research Station, Institute of Urban Environment, Chinese Academy of Sciences, Xiamen, China.

[3] School of Civil & Environmental Engineering and School of Biological Sciences, Georgia Institute of Technology, Atlanta, GA, USA.

[4] State Key Laboratory of Urban and Regional Ecology, Research Center for Eco-Environmental Sciences, Chinese Academy of Sciences, Beijing, China.

[5] Institute of Urban Environment, Chinese Academy of Sciences, Xiamen, China.

Corresponding author: Si-Yu Zhang

E-mail: syzhang@des.ecnu.edu.cn

This PDF file includes:

Supplementary Results

Supplementary Figures 1 to 10

Supplementary Tables 1 to 8

Supplementary Results:

Details of the trained Random Forest classification model

The data used for training were evenly distributed across the 2 groups (NOF:109 samples; OF:109 samples), which ensured that the proportions and samples of each subgroup were consistent in both the training and test sets, which improved the representativeness of the groups. The random forest classification model was built following a two-step training. The first generated random forest classification with the default parameters acquired an error rate of 2.63%, which was used to classify the samples in the test set considering all genera. Mean decrease accuracy (MDA) which indicates the degree of decrease in the predictive accuracy of random forests, that is equal to the contribution of the genus to the classification, was used to estimate the contribution of each genus (Supplementary Table 2). Ten-fold cross-validation was executed with 10 repetitions, to eliminate the undesirable effects of unbalanced division of data in a single division, and to compensate for the limitation of insufficient training data. This approach not only reinforced the stability and reliability of the model's performance estimates but also guaranteed that every sample in the dataset has an equal chance of being included in the test set, reinforcing the representativeness of our training and testing regimes. Supplementary Figure 1 showed the relationship between the cross-validation error rate of the random forest classification model and the number of genera used for fitting. During the second training process, considering both the number of genera and the cross-validation error rate, the top 140 contributing genera (Supplementary Table 2) were selected for training the new random forest classification model using the training set, with optimal parameter adjustment using the R package "caret" (mtry = 2). The error rate of the generated new random forest classification model was 1.32%, which reduced the error rate by half compared to the previous one. Similarly, the test set was used to validate the classification model accuracy, with 31 out of the 33 NOF samples being correctly classified into NOF and all 33 OF samples being correctly classified in OF. The final random forest classification model acquired an accuracy of 0.97, recall of 0.94, and precision of 1. If setting the weights of precision and recall as 1:1, F1 score of 0.97 was obtained, indicating that the constructed random forest classification model can accurately predict the sample groupings.

Estimation of the taxonomic microbial communities and ARGs and MRGs abundances in samples with certain fertilization information and those after grouped using trained Random Forest classification model

Based on the data uploaded or corresponding article information, 109 of the 511 global agricultural soil samples were with certain information as applied with organic fertilizer, and 109 samples were without organic fertilization. The Shannon diversity and composition of microbial community, antibiotic resistance genes (ARGs), Risk ARGs, and metal resistance genes (MRGs) were firstly analyzed only on the 109 identified NOF and 109 identified OF samples, and then compared with the final classified 284 OF and 227 NOF samples by Random Forest classification model.

According to the analysis results of the identified 109 NOF and 109 OF soils, the Shannon diversity was significantly higher in NOF than in OF ($p < 0.001$, Supplementary Fig. 2a). At class level, *Actinomycetia*, *Alphaproteobacteria*, *Betaproteobacteria*, *Gammaproteobacteria*, and *Bacilli* were the dominant bacteria (Supplementary Fig. 2b). Consistently, for the total agriculture soils after grouping based on the Random Forest classification model, the Shannon diversity was also significantly higher in 227 NOF than in 284 OF soils ($p < 0.001$, Fig. 1b). *Actinomycetia*, *Alphaproteobacteria*, *Betaproteobacteria*, *Gammaproteobacteria* and *Bacilli* were the dominant bacteria at the class level in agricultural soils, accounting for 89.5% - 89.7% of the total abundances of microbes (Supplementary Fig. 2d). Slight variances in the relative abundance of these microbes between 227 NOF and 284 OF soils were revealed, i.e., *Actinomycetia* accounting for 35.7% and 31.5%, *Alphaproteobacteria* accounting for 20.9% and 18.7%, *Betaproteobacteria* accounting for 16.3% and 18.5%, *Gammaproteobacteria* accounting for 10.9% and 14.2%, and *Bacilli* accounting for 5.9% and 6.6% in NOF and OF soils, respectively (Supplementary Fig. 2d).

Regarding the abundances and diversities of ARGs, risk ARGs, and MRGs, based on the comparison results of 109 OF and 109 NOF soils with certain fertilization information, the abundance of ARGs was not significantly different in NOF and OF, while the abundance of Risk ARGs and MRGs was significantly higher in OF than in NOF (0.0076 *vs.* 0.0036 and 1.72 *vs.* 1.59 copies per cell, respectively, Supplementary Fig. 2c). A consistent trend was found in the samples after grouping using the Random Forest classification model. The overall relative abundance of ARGs was higher in 284 OF compared to 227 NOF (1.05 *vs.* 0.87 copies per cell) soils but with no statistically

significant differences ($p > 0.05$, Supplementary Fig. 2e). Nevertheless, the relative abundance of Risk ARGs, and MRGs was both significantly ($p < 0.001$) higher in OF than in NOF soils (0.05 *vs.* 0.02 and 2.32 *vs.* 1.46 copies per cell, respectively, Supplementary Fig. 2e). In sum, the overall microbial alpha diversity (Shannon), microbial composition, and relative abundance of ARGs, Risk ARGs, and MRGs trended consistently in NOF and OF before and after Random Forest classification.

Microbial communities in NOF and OF agricultural soils

Microbial interaction networks at the genus level in NOF and OF samples were used to identify significant associations between taxa (Supplementary Fig. 3a, b). The network of OF soils exhibited 91% more edges (2584) between nodes compared to the NOF soils (1352), although number of nodes showed little variances in the network of OF and NOF soils (443 and 413 nodes for OF and NOF, respectively, Supplementary Fig. 3c). This elevation in edge quantity significantly enhanced connection density and yielded more intricate network patterns. Various network metrics such as relative modularity (1.73 in OF and 1.11 in NOF), connectiveness (0.026 in OF and 0.016 in NOF), average clustering coefficient (0.62 in OF and 0.59 in NOF), and average degree (11.67 in OF and 6.55 in NOF; Supplementary Fig. 3c) were revealed with higher values in OF than NOF soils, indicating a heightened complexity in the interaction of microbial communities in agricultural soils with organic fertilizer application.

NMDS plot of overall taxonomic (stress = 0.13, $p = 0.001$) microbial communities showed significantly different cluttering patterns in OF and NOF soils (Supplementary Fig. 3d). NMDS plot of functional (stress = 0.08, $p = 0.001$) microbial communities also showed significantly different cluttering patterns in OF and NOF soils (Supplementary Fig. 3e). Functional genes assigned to protein metabolism were the most dominant functional category of microbes, with average abundances of 70.1 and 77.5 copies per cell in NOF and OF soils, respectively, followed by carbon metabolism (20.7 and 23.4 copies per cell for NOF and OF soils) and amino acids and derivatives (AADs, 18.2 and 23.4 for NOF and OF soils, Supplementary Fig. 3f). Among these dominant functional categories, the relative abundance of carbon metabolism, AADs, cofactors, vitamins, prosthetic groups, pigments (CVPs), miscellaneous, DNA metabolism, clustering-based subsystems (C-bs), regulation and cell signaling (RCs),

cell division and cell cycle (CDCC) was significantly ($p < 0.05$) higher in OF than NOF soils, while the functions associated with phages, prophages and transposable elements (PPTes) was significantly ($p < 0.05$) lower in OF than NOF soils (Supplementary Fig. 3f).

Variant types of ARGs in NOF and OF agricultural soils

A total of 497 ARG subtypes which belonging to 23 ARG types were detected in agricultural soils at the continental level (Supplementary Table 4) and were mostly associated with vancomycin, multidrug, and bacitracin resistance, with average abundances of 0.40, 0.30, and 0.07 copies per cell in OF soils, and 0.62, 0.12, and 0.03 copies per cell in NOF soils (Supplementary Fig. 4). Among them, 323 subtypes were shared by OF and NOF soils and were mostly assigned to beta-lactam (79 subtypes), multidrug (70 subtypes), MLS (40 subtypes), tetracycline (36 subtypes), and aminoglycoside (31 subtypes) resistance genes (Supplementary Table 4). More subtypes of ARGs were detected specifically associated with microbes in OF than NOF soils (131 *vs.* 46; Supplementary Table 4). According to the Wilcoxon test, the relative abundance of aminoglycoside, tetracycline, sulfonamide, chloramphenicol, and kasugamycin resistance genes significantly ($p < 0.001$) increased in OF compared to NOF soils with fold change of 2, 3, 20, 8 and 12. While the relative abundance of vancomycin significantly ($p < 0.001$) increased in NOF than OF soils with a fold change of 0.4 (Supplementary Fig. 4). Among the risk ARG types, the relative abundance of aminoglycoside (mostly comprised of *aadA*, *aph*, *aac* and *ant* genes), multidrug (*mdt* families), chloramphenicol (*cat* families), tetracycline (*tet* families), macrolide-lincosamide-streptogramin (MLS: *erm* families), and quinolone (*qnrB* gene) resistance genes' abundances showed significantly ($p < 0.001$) increased with fold change of 2, 11, 10, 6 and 13 (Supplementary Fig. 4).

Variant types of MRGs in NOF and OF agricultural soils

A total of 316 subtypes of MRGs which belonging to 19 MRG types were detected in these agricultural soils and were mostly shared in NOF and OF agricultural soils (272 MRG subtypes; Supplementary Table 5). A higher number of MRG subtypes was observed in OF than in NOF soils (39 *vs.* 5; Supplementary Table 5). The MRGs

detected in agricultural soils mainly belonged to multimetal resistance genes (87 subtypes), copper (Cu) resistance genes (65 subtypes), and arsenic (As) resistance genes (27 subtypes; Supplementary Table 5). In OF soils, As (abundances of 1.58 copies per cell), multimetal (abundances of 0.26 copies per cell) and chromium (Cr, abundances of 0.14 copies per cell) resistance genes were the most prominent MRG types, while As (abundances of 1.04 copies per cell), iron (Fe, abundances of 0.14 copies per cell), and multimetal (abundances of 0.11 copies per cell) resistance genes were dominated in NOF soils (Supplementary Fig. 4). According to the Wilcoxon test, the relative abundance of As (mostly comprised of *acr3* gene), multimetal, Silver (Ag), Mercury (Hg), Selenium (Se), Cadmium (Cd), Lead (Pb), Tellurium (Te), Gold (Au), Manganese (Mn) was revealed significantly ($p < 0.001$ or 0.01) increased in OF than NOF soil. Among them, the relative abundance of As resistance genes (mostly comprised of *acr3* genes) and multimetal resistance genes (mostly efflux genes) were the most increased with a fold change of 0.52 and 1.34. While the relative abundances of Cr and Cu resistance genes were also increased with fold change of 1.02 and 0.97, their differences in OF *vs.* NOF soils were not statistically significant (Supplementary Fig. 4).

Microbial communities with significantly differential abundances and their association with diverse ARGs and MRGs

According to DESeq2 analysis, a total of 76 genera were detected with significantly increased abundances in OF than NOF soils, while 50 genera were observed with significantly decreased abundances in OF than NOF soils ($p < 0.05$; Supplementary Fig. 6a, Supplementary Table 3). The microbes with statistically significantly higher abundances of log2FoldChange >1 in OF compared to NOF soils were mostly assigned to members of *Actinobacteria*, i.e., *Arthrobacter* (2.49% *vs.* 1.34%), *Phycicoccus* (0.24% *vs.* 0.13%), *Pedococcus* (0.21% *vs.* 0.13%), members of *Bacteroidetes*, i.e., *Flavobacterium* (0.43% *vs.* 0.15%), *Chryseobacterium* (0.24% *vs.* 0.08%), *Pedobacter* (0.10% *vs.* 0.03%), members of *Firmicutes*, i.e., *Clostridium* (0.98% *vs.* 0.06%), and members of *Pseudomonadota*, i.e., *Stenotrophomonas* (1.26% *vs.* 0.64%), *Anaeromyxobacter* (0.87% *vs.* 0.21%), *Xanthomonas* (0.54% *vs.* 0.24%, Supplementary Fig. 5a). In contrast, the abundance of *Metabacillus* (0.04% *vs.* 0.10%, belongs to Firmicutes) and *Pseudomonadota*, mostly comprised of *Porphyrobacter* (0.07% *vs.*

0.16%) and *Tardibacter* (0.05% *vs.* 0.12%) was significantly lower ($p < 0.05$ and log2FoldChange < -1) in OF *vs.* NOF soils (Supplementary Fig. 6a).

Among the microbes with statistically significantly higher or lower abundances in OF *vs.* NOF soils (Supplementary Fig. 6a), the relative abundance of *Flavobacterium*, *Chryseobacterium*, *Pedobacter* (*Bacteroidetes* phylum), and *Clostridium* (*Firmicutes* phylum), and *Stenotrophomonas, Xanthomonas*, *Brevundimonas*, *Agrobacterium* (*Pseudomonadota* phylum) showed a significant ($p < 0.001$ or 0.01) positive correlation with the high abundance and diversity (richness) of the risk ARGs in OF soils (Supplementary Fig. 6b). Consistently, the relative abundance of these microbes also showed a significant ($p < 0.001$ or 0.05) positive correlation with the abundance and diversity (richness) of MRGs in OF soils, except the *Stenotrophomonas* (Supplementary Fig. 6b). The genera affiliated with *Actinobacteria* and *Pseudomonadota* (*Anaeromyxobacter*, *Ideonella*, *Thiobacillus*, *Geobacter*, *Pelobacter*, *Porphyrobacter*, and *Tardibacter*), which acquired significantly variant abundances in OF and NOF soils showed a negative correlation with the risk ARGs and/or MRGs abundance and diversity (Supplementary Fig. 6b). For the functional genes, the abundance of carbon, nitrogen, phosphorus, sulfur, and potassium metabolic genes in agricultural soils showed significantly positive correlations with risk ARGs and MRGs in both NOF and OF agricultural soils ($p < 0.05$, Supplementary Fig. 6c). The richness of ARGs was significantly positively correlated with the most of elemental metabolism genes in the OF soils, while this correlation was detected in the NOF soils only for the carbon and phosphorus metabolism genes (Supplementary Fig. 6c).

Taxonomic classification of AMCCs and their co-existent ARG and MRG subtypes

According to the taxonomic classification of AMCCs, *Pseudomonas* was the primary host where coexisting ARGs and MRGs were found, accounting for 11% and 15% of the total AMCCs in OF and NOF soils, respectively (Supplementary Fig. 7a, b). Other contributing genera included *Aeromonas*, *Arthrobacter*, *Anaeromyxobacter*, *Brevundimonas*, *Pantoea*, *Stenotrophomonas*, *Thiobacillus*, and *Xanthomonas*, which in total represented 69% and 57% of the total AMCCs in OF and NOF soils. The dominant AMCCs taxa corroboratively with the genera that acquired a significantly

increased abundance in OF soils as revealed by DESeq2 analyses (Supplementary Fig. 6a).

The average abundance of AMCCs carrying MGEs, AMCCs carrying virulence factor genes (VFGs), and AMCCs carrying both VFGs and MGEs were all detected with significantly increased abundances in OF compared to NOF soils, which is 0.0202 *vs.* 0.0010, 0.0120 *vs.* 0.0001 and 0.0050 *vs.* 0.0011 copies per cell, respectively (Supplementary Fig. 7c-e). In detail, the most common ARG subtypes coexisting with MRGs were *multidrug_ABC_transporter* (multidrug resistance), *multidrug_transporter* (multidrug resistance), and *rosA* (fosmidomycin resistance), each co-occurring with 18, 14, and 11 MRG subtypes respectively. The MRG subtypes that most frequently coexisted with ARGs were *acrD* (multimetal resistance) and *acn* (Fe resistance), cooccurring with 9 and 8 ARG subtypes, respectively (Fig. 2a).

Taxonomic classification and metabolism pathways of the dominant ARG-MRG-carrying MAGs

Overall, these antibiotic and metal co-resistant bacteria (AMRB) were classified into 12 class, including *Gammaproteobacteria* (48), *Alphaproteobacteria* (12), *Acidobacteriae* (12), *Actinomycetia* (7), *Nitrospirota* (4), *Thermoanaerobaculia* (3), etc, and most of them (68/90) were identified with higher abundance in OF than in NOF agricultural soils (Fig. 5a). These AMRB were mostly resistant to antibiotics including multidrug (84/95), fosmidomycin (30/95), bacitracin (26/95), MLS (9/95), aminoglycoside (7/95), beta-lactam (8/95), tetracycline (8/95), chloramphenicol (7/95), rifamycin (7/95), polymyxin (6/95) and vancomycin (5/95), and metals including Cr (94/95), As (93/95), Fe (81/95), multimetal (77/95), Cu (76/95), Zn (74/95), Se (63/95), Au(32/95) and Ag(21/95). MGEs of these AMRB were classified to the types of recombinase (92/95), transposase (88/95), ICE (82/95), AICE (40/95), IME (34/95), and VFGs of these AMRB were most associated with adherence (94/95), stress survival (84/95), immune modulation (83/95), motility (53/95), biofilm (50/95), regulation (50/95), effector delivery system (49/95), nutritional/metabolic factor (36/95), and antimicrobial activity/competitive advantage (24/95; Fig. 5a).

To further understand the function of AMRB, their elemental cycling functional genes were annotated based on the KEGG database (Supplementary Table 7). The *nirK*

gene was the most frequently occurring functional gene present in 32 AMRB, followed by *pqqC*, *phoD*, and *narG*, present in 31, 27, and 23 AMRB, respectively, and mainly involved in functional processes such as denitrification (NO_2^- to NO), inorganic P dissolution, organic P mineralization, and Nitrate reduction (Supplementary Fig. 9a). OF_MAG84 has the most diverse functional genes (12), followed by NOF_MAG31 (10) and OF_MAG76 (9), all of which are classified as *Gammaproteobacteria* (Supplementary Fig. 9b). Similarly, five AMRB (OF_MAG16, NOF_MAG7, OF_MAG76, NOF_MAG31, and OF_MAG84) belonging to *Gammaproteobacteria* present a complete sulfur-oxidation (SOX) pathway (*soxA*, *soxB*, *soxC*, *soxD*, *soxX*, *soxY*, and *soxZ*) belonging to the sulfur-oxidation bacteria (Supplementary Fig. 9a). Only 13.7% of AMRB (13/95) did not have the functional genes we selected, indicating that they do not have the functions represented by these functional genes or possibly due to the incomplete assembling of the microbial genomes. To sum, most AMRB (82/95) were associated with nitrogen (*narG*, *nirK/S*, and *nosZ*), phosphorus (*phoD* and *pqqC*), or sulfur (*aprA/B*, *dsrB*, *soxA/B/C/D/C/Y/Z*, *dmsA*, and *mddA*) cycling genes, indicating the presence of indigenous functional bacteria (Supplementary Fig. 9a), which also validated previous results showing the positive correlation of functional genes with ARGs and MRGs (Supplementary Fig. 6c).

Soil properties, metagenomic, and metatranscriptomics data profiles of 12 paddy soil samples under different As stress

The soil properties of these 12 samples showed no significant differences, with the total carbon (TC) concentration ranged from 22.00 to 41.00 g kg^{-1}, total nitrogen (TN) concentration ranged from 1.73 to 2.41 g kg^{-1}, total phosphorus (TP) concentration ranged from 0.37 to 1.60 g kg^{-1}, and total sulfate (TS) concentration ranged from 0.19 to 0.49 g kg^{-1}. The concentration of heavy metals besides As was also analyzed with Cr concentrations ranged from 60.3 to 98.02 mg kg^{-1}, Sb concentrations ranged from 1.85 to 3.82 mg kg^{-1}, Cd concentrations ranged from 0.37 to 0.95 mg kg^{-1}, and Cu concentrations ranged from 19.42 to 42.44 mg kg^{-1}. For metagenomic datasets, 10.3 to 13.9 Gbp (12.3 Gbp, on average) of shotgun metagenomic reads and an average read length of 145 bp were acquired for each sample after trimming, and an average of 338,922 contigs and 509,273 ORFs were generated per sample. For metatranscriptomic datasets, an average of 26.7 Gbp (ranging from 24.9 to 29.8 Gbp) shotgun

metatranscriptomic reads and an average read length of 132 bp were obtained for each sample after trimming and removing of rRNA reads.

Defining the high and low risk of global threat potential of AMRB in agricultural soils

The unsupervised learning approach using k-means clustering was employed to categorize the abundance of antibiotic and metal(loid) co-resistant bacteria (AMRB) in 511 agricultural soils into six risk levels. The t-distributed Stochastic Neighbor Embedding (t-SNE) method was applied to visualize the clustering results. According to the result of t-SNE, a significant gap separates the samples into two groups, that is the low-risk group (risk levels 1, 2, and 3) and the high-risk group (risk levels 4, 5, and 6; Supplementary Fig. 10). Regarding the abundance of AMRB, the AMRB abundance in samples categorized into risk levels of 1, 2, and 3 was between 3e-4 to 9e-3 coverage per genome equivalent (CPG). Whereas samples classified into the risk level of 4, 5, and 6 exhibited AMRB abundance of 1e-2 to 1e-1 CPG. Given the clustering results and substantial disparity in AMRB abundance between these groups, we defined samples within risk levels of 1 to 3 as low risk and those with risk levels of 4 to 6 as high risk. The six risk levels therefore served as a critical indicator for differentiating between low and high risks. Upon defining the risk levels, a variety of features (such as climate data, soil properties, etc.) and machine learning algorithms were utilized to predict the microbial risk level for each point at a 0.083° resolution across global farmlands. The predicted risk levels of AMRB in different locations were used to create a map of microbial risk levels across global farmlands, where levels 1-3 were marked as ‘Low Risk’ areas and levels 4-6 were marked as ‘High Risk’ areas.

Supplementary Figures:

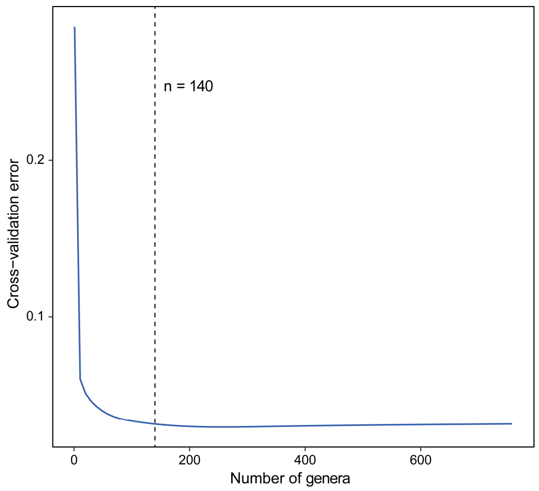

Supplementary Figure 1. **The relationship between Cross-validation error and the number of variables (genera) in the Random Forest (RF) classification model.** Source data were provided as a Source Data file.

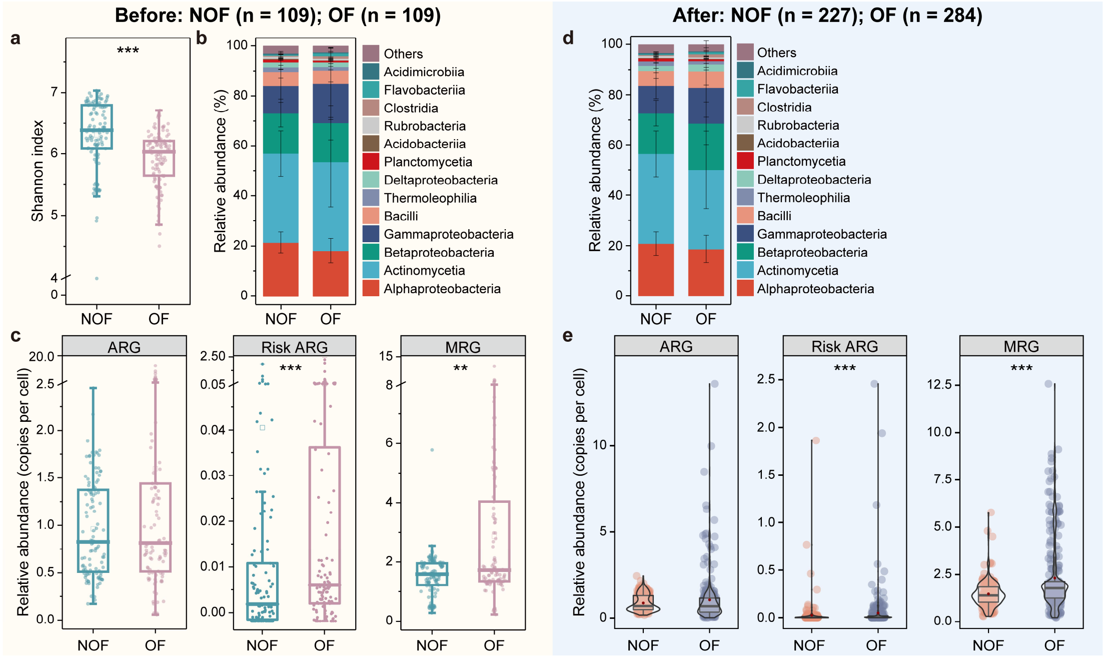

Supplementary Figure 2. **Shannon diversity and composition of microbial community and relative abundance of antibiotic resistance genes (ARGs), Risk ARGs, and metal(loid) resistance genes (MRGs) before (*n* = 109 in NOF and OF) and after (*n* = 227 in NOF and *n* = 284 in OF) grouping using the RF classification model. a** Shannon diversity of microbial community before grouping (Shannon diversity of microbial community after grouping in Figure 1b). **b** Microbial community compositions at the Class level before grouping. **c** Relative abundance of ARGs, Risk ARGs, and MRGs before grouping. **d** Microbial community compositions at the Class level after grouping. **e** Relative abundance of ARGs, Risk ARGs, and MRGs after grouping. The boxes indicated the 25th to 75th percentiles (with the median as a horizontal line), and the whiskers represented the maximum and minimum values except for outliers. Two-sided Wilcoxon test was used for significance analyses (**$p < 0.01$; ***$p < 0.001$). The boxes indicated the 25th to 75th percentiles (with the median as a horizontal line), and the whiskers represented the maximum and minimum values except for outliers. Each point was a sample in box plot. In Supplementary Figure 2b and 2d, each error bar corresponds to the standard deviation (SD) and data were showed as mean ± SD. Source data were provided as a Source Data file.

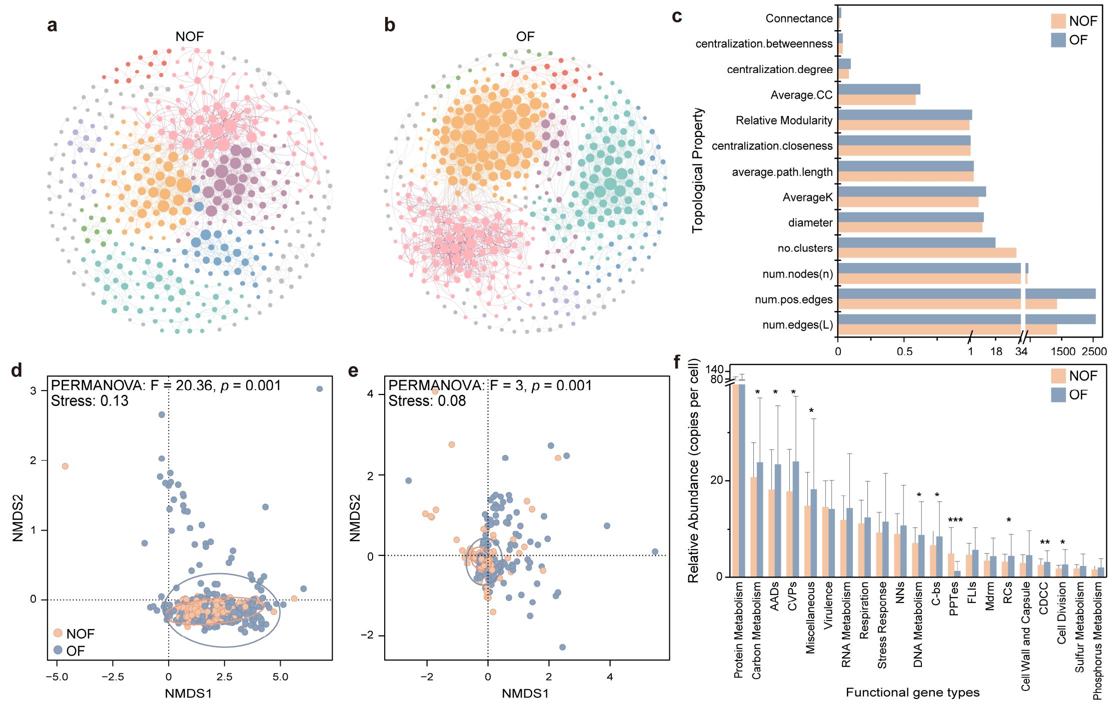

Supplementary Figure 3. **Microbial interaction networks, beta-diversity of microbial composition and functional gene composition, and relative abundance of functional gene types.** Networks of genus level based on Spearman analysis of NOF samples **(a)** and OF samples **(b)**. Network colored by modularity class. In each network, node size was proportional to the number of connections (i.e., degree); edge thickness was proportional to Spearman's r. **c** Comparison of network topology of NOF and OF. **d** Beta-diversity of microbial community composition (PERMANOVA, F = 20.36, p = 0.001). **e** Beta-diversity of functional gene composition (PERMANOVA, F = 3, p = 0.001). **f** Relative abundance of functional gene types (Top 20) according to the SEED level 1 classifications (two-sides Wilcoxon test, *$p < 0.05$; **$p < 0.01$; ***$p < 0.001$). Each error bar corresponds to the SD and data were showed as mean + SD. Source data were provided as a Source Data file. Average.CC: average clustering coefficient; AverageK: average degree; AADs: Amino Acids and Derivatives; CVPs: Cofactors, Vitamins, Prosthetic Groups, Pigments; NNs: Nucleosides and Nucleotides; C-bs: Clustering-based subsystems; PPTes: Phages, Prophages, Transposable elements; FLIs: Fatty Acids, Lipids, and Isoprenoids; Mdrm: Metabolite damage and its repair or mitigation; RCs: Regulation and Cell signaling; CDCC: Cell Division and Cell Cycle.

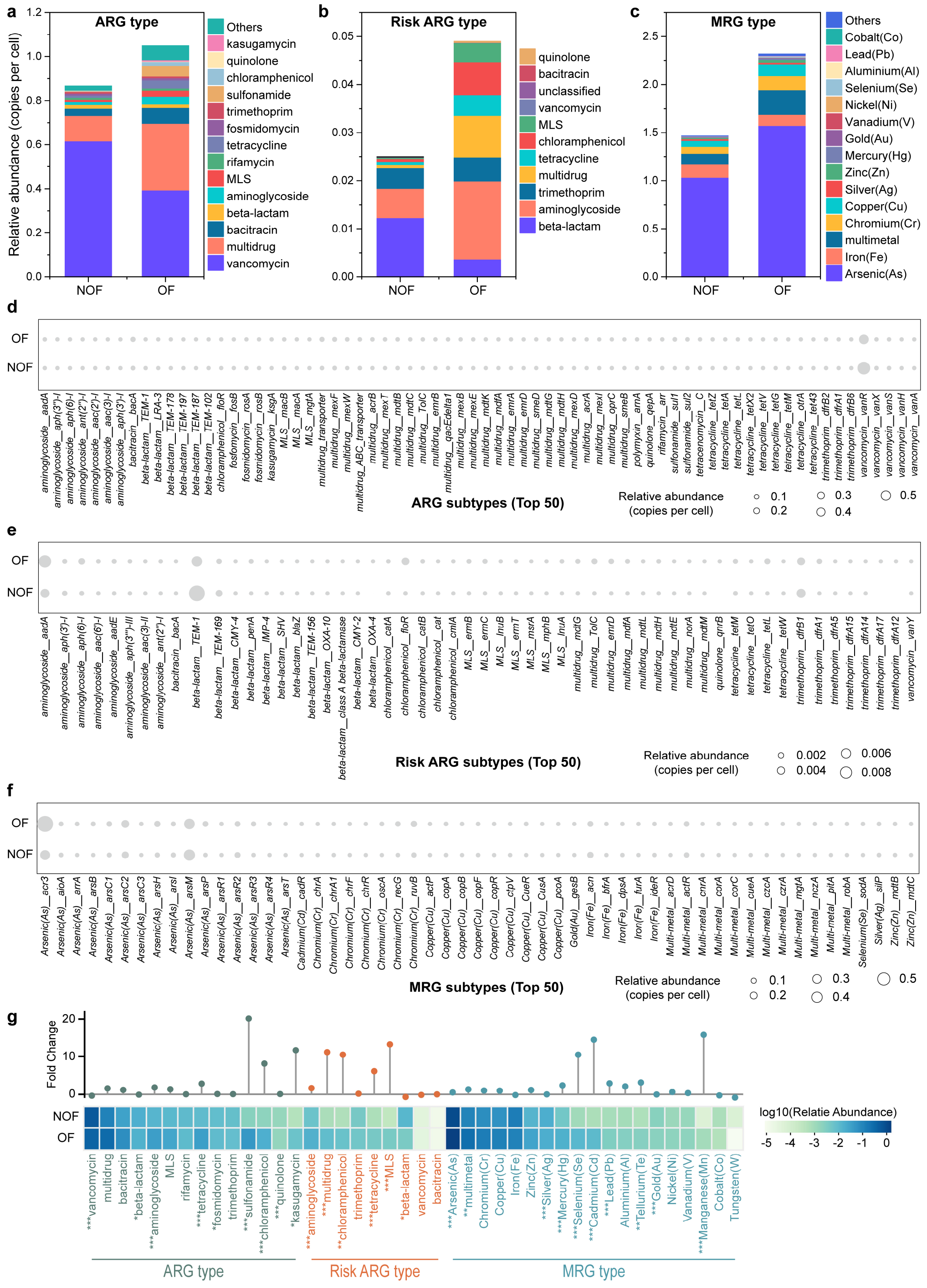

Supplementary Figure 4. **The relative abundance of types and subtypes of ARGs, Risk ARGs, and MRGs.** The relative abundance of ARG types **(a)**, Risk ARG types **(b)**, and MRG types **(c)** in NOF and OF samples. The relative abundance of the top 50 ARG subtypes **(d)**, Risk ARG subtypes **(e)**, and MRG subtypes **(f)**. **g** The fold changes of relative abundance of major ARG types, Risk ARG types, and MRG types (two-sides Wilcoxon test, **p* < 0.05; ***p* < 0.01; ****p* < 0.001). Different colors represented

different gene types. Source data were provided as a Source Data file. MLS: macrolide-lincosamide-streptogramin.

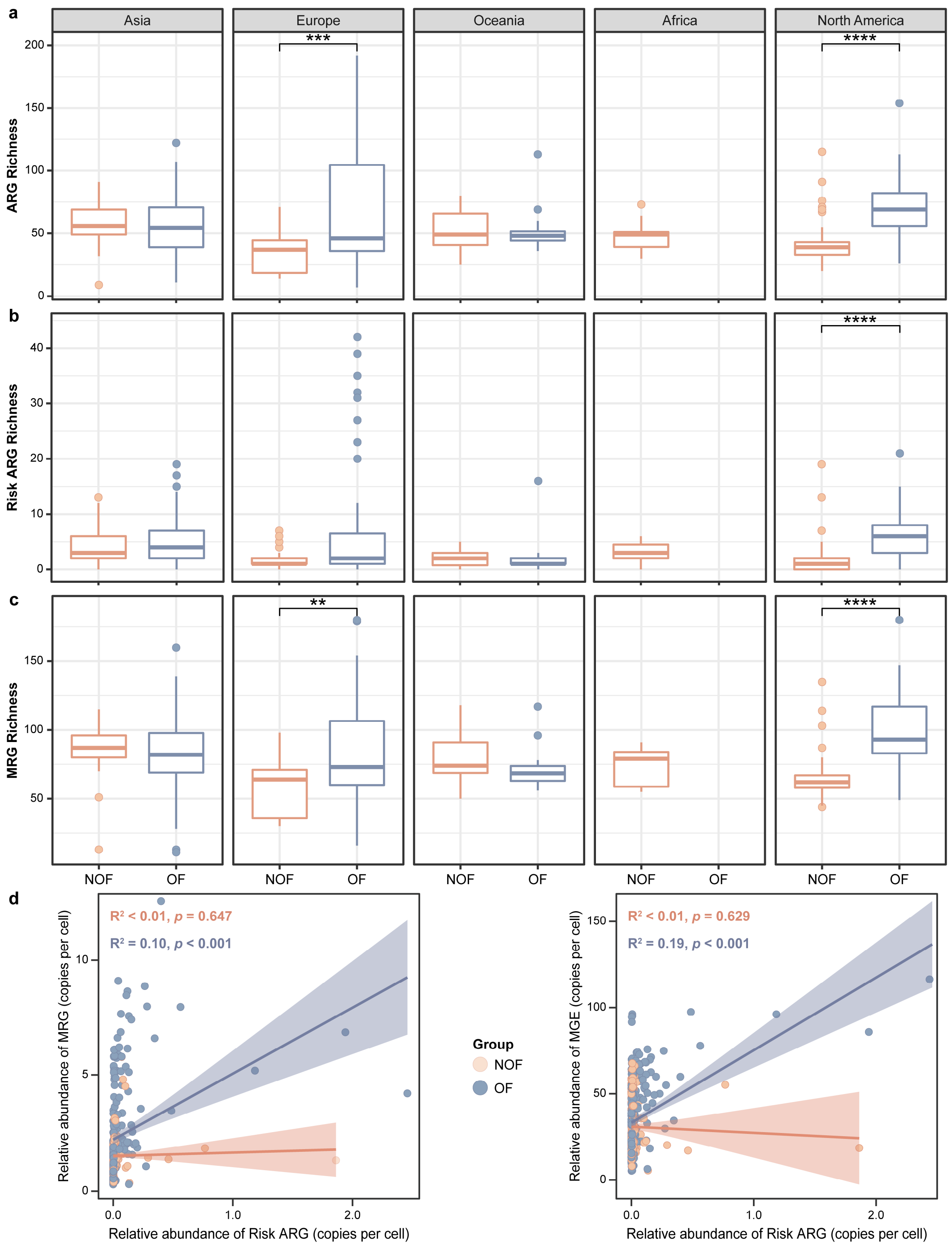

Supplementary Figure 5. **The diversity (Richness) of resistance genes on different continents.** Comparison of Richness of ARGs **(a)**, Risk ARGs **(b)**, and MRGs **(c)** on different continents between NOF and OF (two-sides Wilcoxon test, $**p < 0.01$; $***p < 0.001$; $****p < 0.0001$). The boxes indicated the 25th to 75th percentiles (with the median as a horizontal line), and the whiskers represented the maximum and minimum values except for outliers. Single points represented outliers. **d** Linear regression analysis of relative abundance between Risk ARGs and MRGs/mobile genetic elements (MGEs). Orange and blue dots represented NOF and OF samples, respectively. Source

data were provided as a Source Data file. ARG: antibiotic resistance genes; MRG: metal(loid) resistance gene.

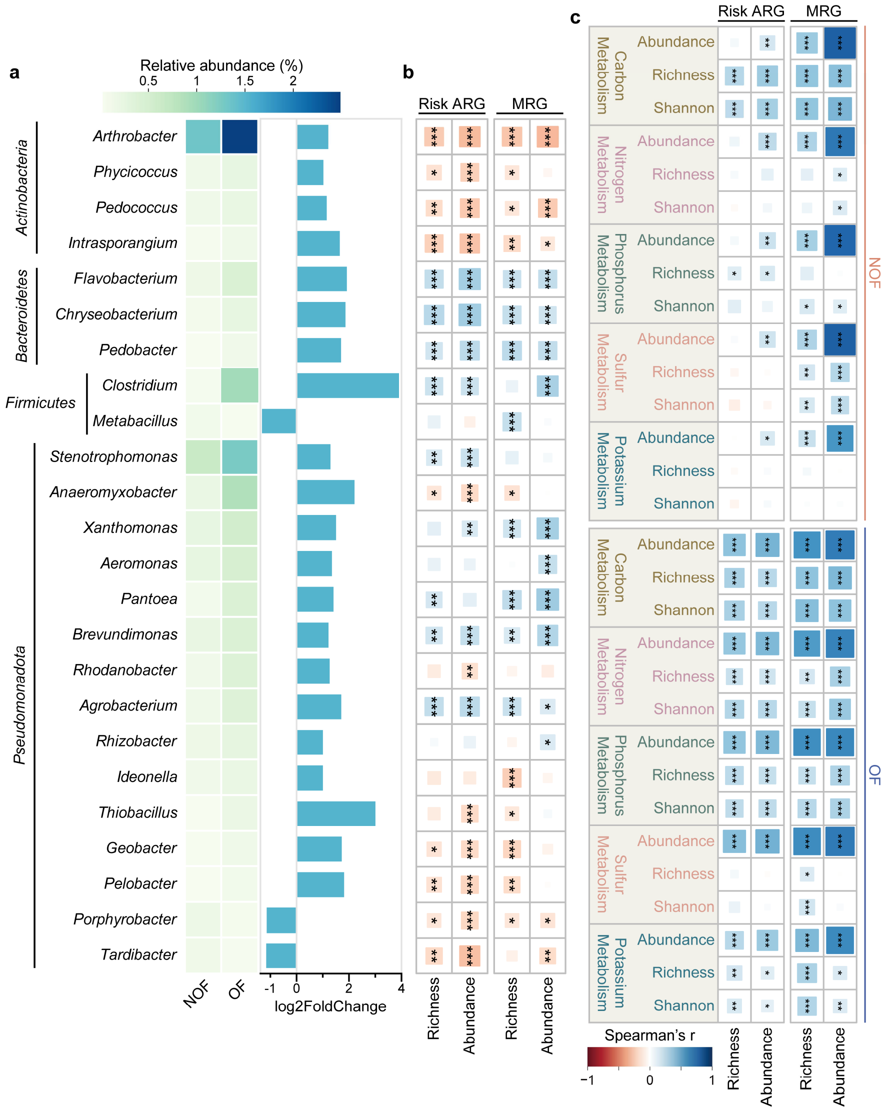

Supplementary Figure 6. **Genera with significant variation between NOF and OF and their association with Richness and relative abundance of Risk ARGs and MRGs. a** Genera (relative abundance > 0.1%) with significant differences in relative abundance between NOF and OF (DESeq2, |log2FoldChange| ≥ 1, adjusted $p < 0.05$). **b** The Spearman correlation (*$p < 0.05$; **$p < 0.01$; ***$p < 0.001$) between the relative abundance of genera and the relative abundance and diversity (Richness) of Risk ARGs and MRGs in OF samples. **c** The Spearman correlation (*$p < 0.05$; **$p < 0.01$; ***$p < 0.001$) between the relative abundance of carbon (yellow), nitrogen(pink), phosphorus(green), sulfur(orange), and potassium (blue) metabolic genes and the relative abundance and diversity (Richness) of Risk ARGs and MRGs. Source data were provided as a Source Data file. ARG: antibiotic resistance gene; MRG: metal(loid) resistance gene.

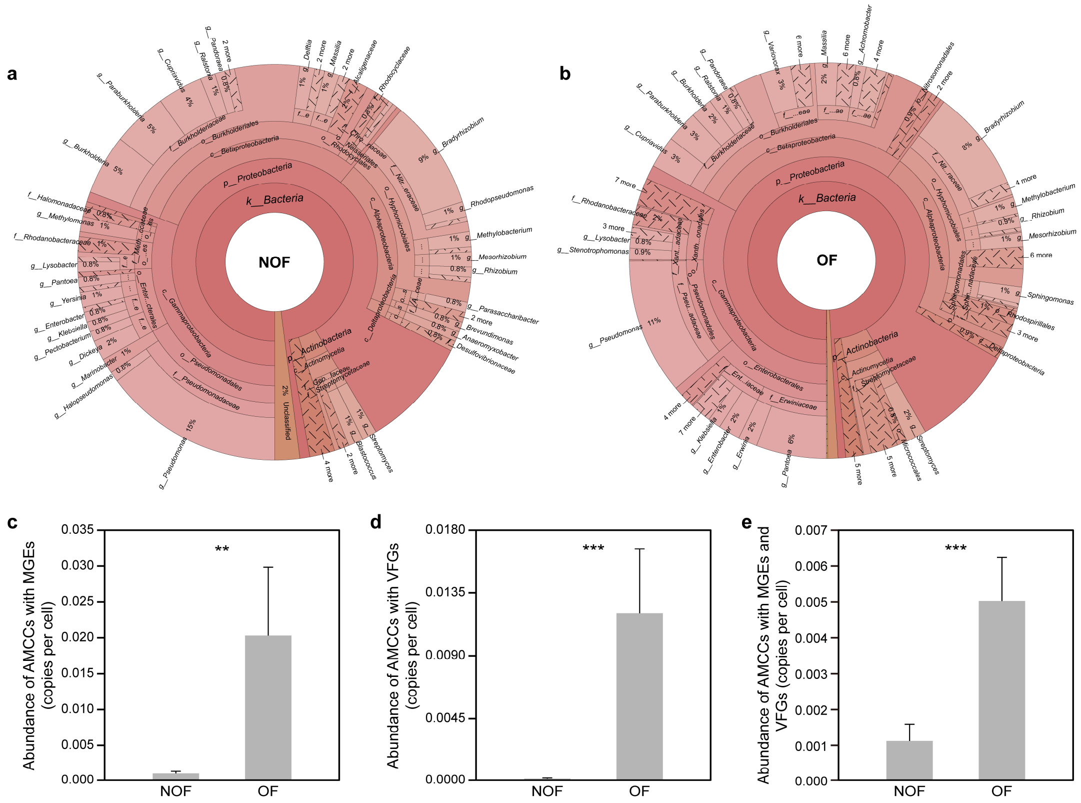

Supplementary Figure 7. **Taxonomic classification and abundance of ARG-MRG-carrying contigs (AMCCs).** Taxonomic classification of AMCCs in NOF **(a)** and OF **(b)**. Abundance of AMCCs with MGEs **(c)**, virulence factor genes (VFGs) **(d)**, and MGEs and VFGs **(e)** in NOF and OF (two-sides Wilcoxon test, **p < 0.01; ***p < 0.001). Each error bar corresponds to the SD and data were showed as mean + SD. Source data were provided as a Source Data file. MGE: mobile genetic element.

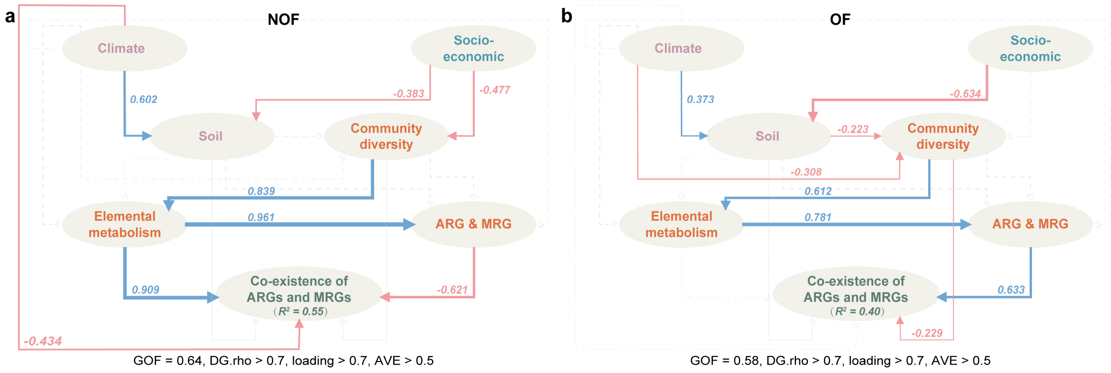

Supplementary Figure 8. **Partial Least Square- Structural Equation Modelling (PLS-SEM) showing the direct and indirect effects of different factors on the co-existence of ARGs and MRGs for NOF (a) and OF (b) samples.** Blue, red, and gray colors indicate significantly positive, significantly negative, and no significant effects, respectively. Path coefficients and coefficients of determination (R^2) were calculated after bootstraps (n = 100), and all path coefficients shown were statistically significant ($p < 0.05$). Different colors represented different types of variables. Source data were provided as a Source Data file. ARG: antibiotic resistance gene; MRG: metal(loid) resistance gene; GOF: goodness of fit; DG.rho: Dillon-Goldstein's rho; AVE: average variance extracted.

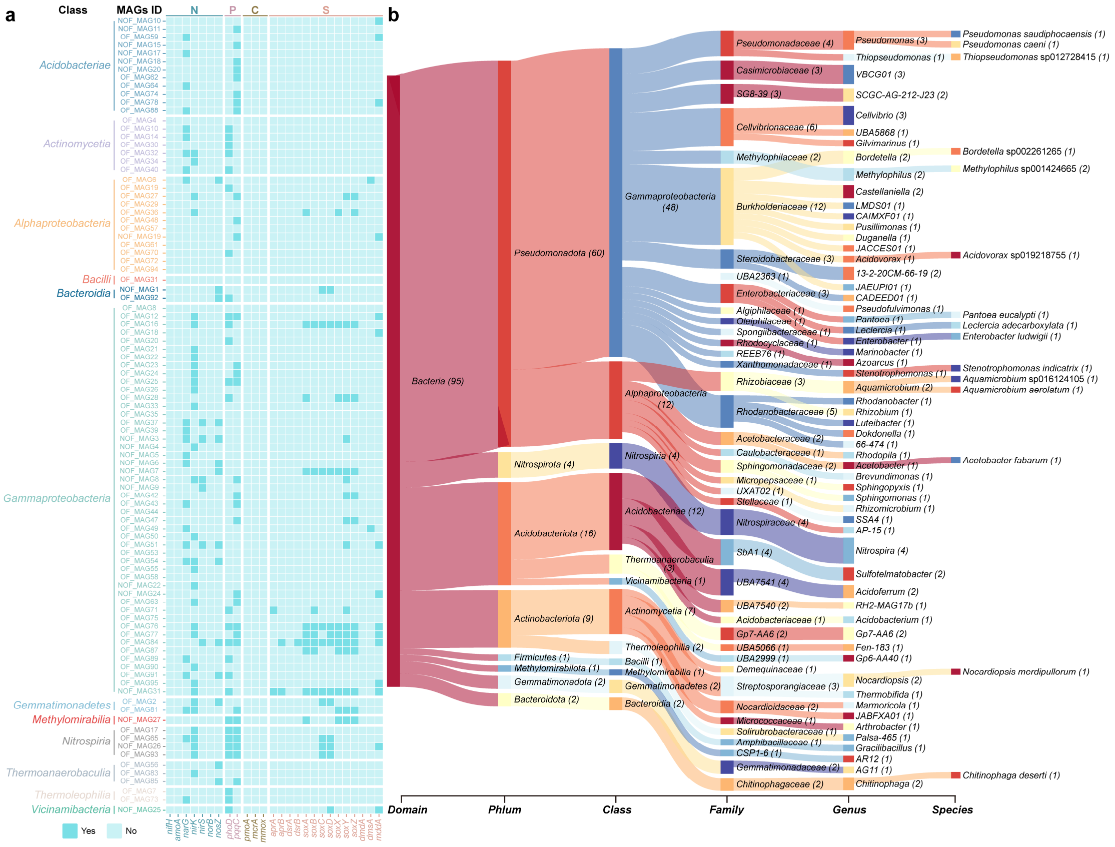

Supplementary Figure 9. **Annotation of elemental cycling functional genes and taxonomic classification of metagenome-assembled genomes (MAGs). a** The presence or absence of carbon (yellow), nitrogen(blue), phosphorus(pink), sulfur(orange) cycling functional genes in 95 MAGs containing AMCCs (see Supplementary Table 7). The colors of MAGs represented the different Class levels to which these MAGs were assigned. **b** Taxonomic classification of 95 MAGs containing AMCCs. Source data were provided as a Source Data file.

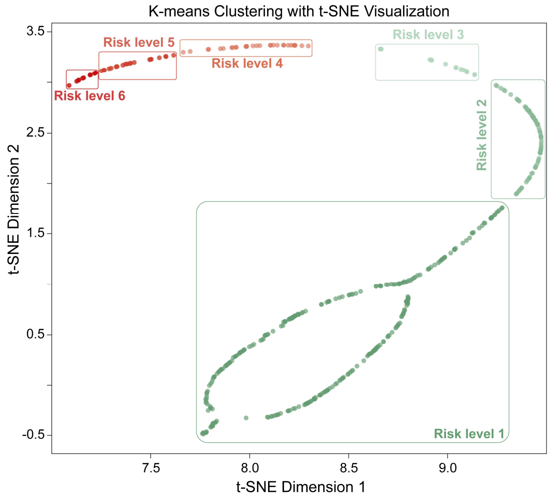

Supplementary Figure 10. **Visualization of antibiotic and metal(loid) co-resistant bacteria (AMRB) abundance clusters in agricultural soil samples using t-distributed stochastic neighbor embedding (t-SNE).** Each point represents an individual soil sample, color-coded according to its assigned risk level based on the k-means method. Source data were provided as a Source Data file.

Supplementary Tables:

Supplementary Table 1. **Detailed information on global agricultural soil samples (n = 511).**

Accession Number	Sample ID	Group	Continent	Country	Longitude	Latitude
SRR13062006	NOF_110	NOF	Africa	South Africa	26.5296	-25.9946
SRR13062007	NOF_119	NOF	Africa	South Africa	26.5296	-25.9946
SRR13062008	NOF_120	NOF	Africa	South Africa	26.5296	-25.9946
SRR13062099	NOF_121	NOF	Africa	South Africa	27.33	-26.11
SRR13062098	NOF_122	NOF	Africa	South Africa	27.33	-26.11
SRR13062097	NOF_123	NOF	Africa	South Africa	27.33	-26.11
SRR5512132	OF_97	OF	Asia	China	130.2125	46.40667
SRR5512133	OF_161	OF	Asia	China	131.69722	46.74472
SRR5512134	OF_168	OF	Asia	China	120.05583	31.9375
SRR5512135	OF_178	OF	Asia	China	119.50972	30.91778
SRR5512136	OF_189	OF	Asia	China	102.74125	23.1295
SRR5512137	OF_190	OF	Asia	China	102.38253	23.21189
SRR5512138	OF_198	OF	Asia	China	101.03506	23.05222
SRR5512139	OF_204	OF	Asia	China	101.24619	24.47039
SRR5512140	OF_213	OF	Asia	China	133.46556	48.04444
SRR5512141	OF_98	OF	Asia	China	131.16583	45.28139
SRR5512142	OF_108	OF	Asia	China	114.11722	30.90472
SRR5512143	OF_119	OF	Asia	China	113.28444	30.38
SRR5512144	OF_130	OF	Asia	China	112.34306	28.79583
SRR5512145	OF_132	OF	Asia	China	113.8225	29.95
SRR5512146	OF_140	OF	Asia	China	121.24667	30.70694
SRR5512147	OF_148	OF	Asia	China	120.23528	30.63278
SRR19631180	OF_162	OF	Asia	China	109.21	27.52
SRR19631181	OF_163	OF	Asia	China	109.21	27.52

SRR19631182	OF_164	OF	Asia	China	109.21	27.52
SRR17477666	OF_165	OF	Asia	China	-	-
SRR17477667	OF_166	OF	Asia	China	-	-
SRR17477668	OF_167	OF	Asia	China	-	-
SRR17477669	NOF_140	NOF	Asia	China	-	-
SRR17477670	NOF_141	NOF	Asia	China	-	-
SRR14660885	OF_169	OF	Asia	China	-	-
SRR14660886	OF_170	OF	Asia	China	-	-
SRR14660887	NOF_142	NOF	Asia	China	-	-
SRR14660888	OF_171	OF	Asia	China	-	-
SRR14660889	OF_172	OF	Asia	China	-	-
SRR14660890	OF_173	OF	Asia	China	-	-
SRR14660891	OF_174	OF	Asia	China	-	-
SRR14660892	OF_175	OF	Asia	China	-	-
SRR14660893	OF_176	OF	Asia	China	-	-
SRR14660894	OF_177	OF	Asia	China	-	-
SRR14660895	OF_179	OF	Asia	China	-	-
SRR14660896	OF_180	OF	Asia	China	-	-
SRR14292197	OF_181	OF	Asia	China	104.06666	30.66667
SRR14292196	OF_182	OF	Asia	China	104.06666	30.66667
SRR14292189	OF_183	OF	Asia	China	104.06666	30.66667
SRR14292188	OF_184	OF	Asia	China	104.06666	30.66667
SRR14292187	OF_185	OF	Asia	China	106.71666	26.26666
SRR14292186	OF_186	OF	Asia	China	106.71666	26.26666
SRR14292185	OF_187	OF	Asia	China	106.71666	26.26666
SRR14292184	OF_188	OF	Asia	China	106.71666	26.26666
SRR13336287	NOF_18	NOF	Asia	China	108.083	34.03
SRR13336334	NOF_19	NOF	Asia	China	108.083	34.03
SRR13336326	NOF_20	NOF	Asia	China	108.083	34.03
SRR13336319	NOF_21	NOF	Asia	China	108.083	34.03

SRR13336311	NOF_22	NOF	Asia	China	108.083	34.03
SRR13336303	NOF_23	NOF	Asia	China	108.083	34.03
SRR13336296	NOF_24	NOF	Asia	China	108.083	34.03
SRR13336288	NOF_25	NOF	Asia	China	108.083	34.03
SRR11823718	NOF_143	NOF	Asia	China	86.02	44.18
SRR11823721	NOF_144	NOF	Asia	China	86.02	44.18
SRR11823722	NOF_145	NOF	Asia	China	86.02	44.18
CRR325346	OF_191	OF	Asia	China	-	-
CRR325348	OF_192	OF	Asia	China	-	-
SRR15167835	OF_193	OF	Asia	China	118.038	24.6389
SRR15167834	OF_194	OF	Asia	China	118.038	24.6389
SRR15167830	OF_195	OF	Asia	China	117.939	24.58
SRR15167829	OF_196	OF	Asia	China	117.939	24.58
SRR15167851	OF_197	OF	Asia	China	117.939	24.58
SRR13614401	NOF_146	NOF	Asia	China	116.65	37.3
SRR13614390	NOF_147	NOF	Asia	China	116.65	37.3
SRR13614402	OF_199	OF	Asia	China	116.65	37.3
SRR11450581	OF_200	OF	Asia	China	112.685873	28.171707
SRR11450582	OF_201	OF	Asia	China	112.760733	28.116708
SRR11450583	OF_202	OF	Asia	China	112.913319	28.176739
DRR012573	OF_203	OF	Asia	Japan	-	-
SRR13617630	OF_205	OF	Asia	India	94.1965	26.7248
SRR13617631	OF_206	OF	Asia	India	94.1965	26.7248
DRR160703	OF_207	OF	Asia	Japan	130.766	32.889
DRR160704	OF_208	OF	Asia	Japan	130.766	32.889
DRR160705	OF_209	OF	Asia	Japan	130.766	32.889
DRR160706	OF_210	OF	Asia	Japan	130.766	32.889
DRR160707	OF_211	OF	Asia	Japan	130.766	32.889
DRR160708	OF_212	OF	Asia	Japan	130.766	32.889
DRR160709	OF_214	OF	Asia	Japan	139.539	35.735

DRR160710	OF_215	OF	Asia	Japan	139.539	35.735
DRR160711	OF_216	OF	Asia	Japan	139.539	35.735
DRR160712	OF_217	OF	Asia	Japan	139.539	35.735
DRR160713	OF_218	OF	Asia	Japan	139.539	35.735
DRR160714	OF_219	OF	Asia	Japan	139.539	35.735
DRR160715	OF_220	OF	Asia	Japan	140.244	38.249
DRR160716	OF_221	OF	Asia	Japan	140.244	38.249
DRR160717	OF_222	OF	Asia	Japan	140.244	38.249
DRR160718	OF_223	OF	Asia	Japan	140.244	38.249
DRR160719	OF_99	OF	Asia	Japan	140.244	38.249
DRR160720	OF_100	OF	Asia	Japan	140.244	38.249
SRR2070858	OF_101	OF	Asia	Vietnam	140.244	38.249
SRR5823604	OF_224	OF	Europe	Italy	8.25	45.19
SRR5823605	OF_234	OF	Europe	Italy	8.25	45.19
SRR5823606	OF_241	OF	Europe	Italy	8.25	45.19
SRR5823607	OF_242	OF	Europe	Italy	8.25	45.19
SRR5823608	OF_250	OF	Europe	Italy	8.25	45.19
SRR5823609	OF_252	OF	Europe	Italy	8.25	45.19
SRR5823611	OF_253	OF	Europe	Italy	8.25	45.19
SRR17938967	OF_254	OF	Europe	Russian Federation	83.47	55.01
SRR17938968	OF_255	OF	Europe	Russian Federation	83.47	55.01
SRR17938975	OF_225	OF	Europe	Russian Federation	83.47	55.01
SRR17938976	OF_226	OF	Europe	Russian Federation	83.47	55.01
SRR17938977	OF_227	OF	Europe	Russian Federation	83.47	55.01
SRR17938978	OF_228	OF	Europe	Russian Federation	83.47	55.01
SRR17938979	OF_229	OF	Europe	Russian Federation	83.47	55.01
SRR17938980	OF_230	OF	Europe	Russian Federation	83.47	55.01
ERR7672969	NOF_148	NOF	Europe	The United Kingdom	-1.605268	52.205322
ERR7672970	OF_231	OF	Europe	The United Kingdom	-1.605268	52.205322
ERR7672971	OF_232	OF	Europe	The United Kingdom	-1.605268	52.205322

ERR7672972	OF_233	OF	Europe	The United Kingdom	-1.605268	52.205322
ERR3929365	OF_235	OF	Europe	Switzerland	7.233683	46.931584
ERR3943989	OF_236	OF	Europe	Switzerland	7.233683	46.931584
ERR3929366	OF_237	OF	Europe	Switzerland	7.233683	46.931584
ERR3943990	OF_238	OF	Europe	Switzerland	7.233683	46.931584
ERR3929373	OF_239	OF	Europe	Switzerland	7.233683	46.931584
ERR3943997	OF_240	OF	Europe	Switzerland	7.233683	46.931584
ERR2486618	NOF_149	NOF	Europe	Finland	21.689	60.746
ERR2486619	NOF_150	NOF	Europe	Finland	21.689	60.746
ERR2486620	NOF_151	NOF	Europe	Finland	21.689	60.746
ERR2486621	NOF_152	NOF	Europe	Finland	21.689	60.746
ERR2486622	NOF_153	NOF	Europe	Finland	21.689	60.746
ERR2486623	NOF_154	NOF	Europe	Finland	21.689	60.746
ERR2486624	NOF_155	NOF	Europe	Finland	22.64	60.559
ERR2486625	NOF_156	NOF	Europe	Finland	22.64	60.559
ERR2486626	NOF_157	NOF	Europe	Finland	22.64	60.559
ERR2486627	NOF_158	NOF	Europe	Finland	22.642	60.561
ERR2486628	NOF_159	NOF	Europe	Finland	22.642	60.561
ERR2486629	NOF_160	NOF	Europe	Finland	22.642	60.561
ERR2486630	NOF_161	NOF	Europe	Finland	22.992	60.426
ERR2486631	NOF_162	NOF	Europe	Finland	22.992	60.426
ERR2486632	NOF_163	NOF	Europe	Finland	22.992	60.426
ERR2486633	OF_243	OF	Europe	Finland	23	60.425
ERR2486634	OF_244	OF	Europe	Finland	23	60.425
ERR2486635	NOF_164	NOF	Europe	Finland	23	60.425
SRR9733659	OF_245	OF	Europe	Slovenia	15.04	46.03
SRR9733672	OF_246	OF	Europe	Slovenia	15.04	46.03
SRR9733674	OF_247	OF	Europe	Switzerland	8.01	47.3
SRR7013867	NOF_167	NOF	North America	Canada	-107.8	50.28
SRR7013889	NOF_171	NOF	North America	Canada	-107.8	50.28

SRR7013903	NOF_176	NOF	North America	Canada	-107.8	50.28
SRR7013874	NOF_182	NOF	North America	Canada	-107.8	50.28
SRR7013894	NOF_183	NOF	North America	Canada	-107.8	50.28
SRR7013900	NOF_191	NOF	North America	Canada	-107.8	50.28
SRR7013862	NOF_202	NOF	North America	Canada	-107.8	50.28
SRR7013873	NOF_208	NOF	North America	Canada	-107.8	50.28
SRR7013901	NOF_218	NOF	North America	Canada	-107.8	50.28
SRR7013893	NOF_168	NOF	North America	Canada	-107.8	50.28
SRR14458730	NOF_169	NOF	North America	The United States	-119.518373	36.597906
SRR14458731	NOF_170	NOF	North America	The United States	-119.518373	36.597906
SRR14458735	NOF_172	NOF	North America	The United States	-119.518373	36.597906
SRR14458736	NOF_173	NOF	North America	The United States	-119.518373	36.597906
SRR14458739	NOF_174	NOF	North America	The United States	-119.518373	36.597906
SRR14458740	NOF_175	NOF	North America	The United States	-119.518373	36.597906
ERR1939266	NOF_177	NOF	North America	The United States	-88.242	40.075
ERR1939268	NOF_178	NOF	North America	The United States	-88.242	40.075
ERR1939270	NOF_179	NOF	North America	The United States	-88.242	40.075
ERR1939272	NOF_180	NOF	North America	The United States	-88.242	40.075
SRR5687767	NOF_181	NOF	North America	The United States	-111.8143	41.7655
SRR5689334	OF_256	OF	North America	The United States	-83.4195	33.8834
SRR16123966	OF_257	OF	North America	The United States	-121.875957	38.545045
SRR16123968	OF_258	OF	North America	The United States	-121.871302	38.540587
SRR16123972	OF_259	OF	North America	The United States	-121.870682	38.540611
SRR16123992	OF_260	OF	North America	The United States	-121.874259	38.543123
SRR16123995	OF_261	OF	North America	The United States	-121.874896	38.543092
SRR16124002	OF_262	OF	North America	The United States	-121.876575	38.545011
SRR8987900	NOF_184	NOF	North America	Mexico	-109.55	27.22
SRR8987901	NOF_185	NOF	North America	Mexico	-109.55	27.22
SRR5678809	NOF_186	NOF	North America	The United States	-92.5	43
SRR5678810	NOF_187	NOF	North America	The United States	-92.5	43

SRR5260274	NOF_51	NOF	North America	The United States	-121.653	38.1087
SRR5260273	NOF_52	NOF	North America	The United States	-121.653	38.1087
SRR5260272	NOF_54	NOF	North America	The United States	-121.653	38.1087
SRR12823098	NOF_188	NOF	North America	The United States	-119.74	46.25
SRR12823099	NOF_189	NOF	North America	The United States	-119.74	46.25
SRR12823100	NOF_190	NOF	North America	The United States	-119.74	46.25
SRR12823102	NOF_192	NOF	North America	The United States	-119.74	46.25
SRR12823103	NOF_193	NOF	North America	The United States	-119.74	46.25
SRR12823106	NOF_194	NOF	North America	The United States	-119.74	46.25
SRR12823107	NOF_195	NOF	North America	The United States	-119.74	46.25
SRR12823109	NOF_196	NOF	North America	The United States	-119.74	46.25
SRR12823110	NOF_197	NOF	North America	The United States	-119.74	46.25
SRR12823111	NOF_198	NOF	North America	The United States	-119.74	46.25
SRR12823114	NOF_199	NOF	North America	The United States	-119.74	46.25
SRR12823117	NOF_200	NOF	North America	The United States	-119.74	46.25
SRR12823119	NOF_201	NOF	North America	The United States	-119.74	46.25
SRR6435953	OF_263	OF	North America	The United States	-79.05	35.91
SRR1926159	NOF_101	NOF	Oceania	Australia	147.178	-19.733
SRR1926199	NOF_102	NOF	Oceania	Australia	147.178	-19.733
SRR1926200	NOF_103	NOF	Oceania	Australia	147.178	-19.733
SRR1926201	NOF_104	NOF	Oceania	Australia	147.178	-19.733
SRR3591800	NOF_225	NOF	Oceania	Australia	152.379908	-25.025508
SRR3592089	OF_283	OF	Oceania	Australia	152.379908	-25.025508
SRR3592090	NOF_226	NOF	Oceania	Australia	152.379908	-25.025508
SRR3592108	OF_284	OF	Oceania	Australia	152.379908	-25.025508
ERR3029102	NOF_227	NOF	Oceania	Australia	138.6904631	-34.5376142
ERR3029103	NOF_222	NOF	Oceania	Australia	138.6904631	-34.5376142
ERR3029104	NOF_223	NOF	Oceania	Australia	138.6904631	-34.5376142
ERR3029105	NOF_224	NOF	Oceania	Australia	138.6904631	-34.5376142
SRR3601979	OF_275	OF	Oceania	Australia	152.38	-25.03

SRR3601981	OF_276	OF	Oceania	Australia	152.38	-25.03
SRR3601982	OF_277	OF	Oceania	Australia	152.38	-25.03
SRR3601984	OF_278	OF	Oceania	Australia	152.38	-25.03
SRR3601985	OF_279	OF	Oceania	Australia	152.38	-25.03
SRR3601987	OF_280	OF	Oceania	Australia	152.38	-25.03
SRR3601989	OF_281	OF	Oceania	Australia	152.38	-25.03
SRR5086369/SRR5097182	OF_282	OF	Oceania	Australia	152.38	-25.03
SRR1190384	OF_1	OF	Asia	China	111.446	28.929
SRR1190383	OF_3	OF	Asia	China	111.446	28.929
SRR1190350	OF_13	OF	Asia	China	111.446	28.929
SRR1190349	OF_19	OF	Asia	China	111.446	28.929
SRR1190336	OF_27	OF	Asia	China	120.7808	30.639
SRR1190334	OF_30	OF	Asia	China	120.7808	30.639
SRR1190316	OF_36	OF	Asia	China	116.933	28.206
SRR1190311	OF_38	OF	Asia	China	116.933	28.206
SRR1190308	OF_39	OF	Asia	China	110.0736	20.566
SRR1190306	OF_2	OF	Asia	China	110.0736	20.566
SRR11823711	NOF_1	NOF	Asia	China	86.02	44.18
SRR11823712	NOF_2	NOF	Asia	China	86.02	44.18
SRR11823713	NOF_3	NOF	Asia	China	86.02	44.18
SRR11823714	NOF_4	NOF	Asia	China	86.02	44.18
SRR11823715	NOF_5	NOF	Asia	China	86.02	44.18
SRR11823716	NOF_6	NOF	Asia	China	86.02	44.18
SRR11823717	NOF_7	NOF	Asia	China	86.02	44.18
SRR11823719	NOF_8	NOF	Asia	China	86.02	44.18
SRR11823720	NOF_9	NOF	Asia	China	86.02	44.18
SRR19174815	OF_4	OF	Asia	China	-	-
SRR19174816	OF_5	OF	Asia	China	-	-
SRR19174817	OF_6	OF	Asia	China	-	-
SRR19174818	OF_7	OF	Asia	China	-	-

SRR19174819	OF_8	OF	Asia	China	-	-
SRR19174820	OF_9	OF	Asia	China	-	-
SRR13614383	OF_10	OF	Asia	China	116.65	37.3
SRR13614385	OF_11	OF	Asia	China	116.65	37.3
SRR13614384	OF_12	OF	Asia	China	116.65	37.3
SRR13614381	NOF_10	NOF	Asia	China	116.65	37.3
SRR13614380	NOF_11	NOF	Asia	China	116.65	37.3
SRR13614382	NOF_12	NOF	Asia	China	116.65	37.3
SRR13614399	NOF_13	NOF	Asia	China	116.65	37.3
SRR13614379	NOF_14	NOF	Asia	China	116.65	37.3
SRR13614400	NOF_15	NOF	Asia	China	116.65	37.3
SRR13614397	OF_14	OF	Asia	China	116.65	37.3
SRR13614396	OF_15	OF	Asia	China	116.65	37.3
SRR13614398	OF_16	OF	Asia	China	116.65	37.3
SRR13614395	OF_17	OF	Asia	China	116.65	37.3
SRR13614394	OF_18	OF	Asia	China	116.65	37.3
SRR13614393	OF_20	OF	Asia	China	116.65	37.3
SRR13614392	OF_21	OF	Asia	China	116.65	37.3
SRR13614391	OF_22	OF	Asia	China	116.65	37.3
SRR13614389	OF_23	OF	Asia	China	116.65	37.3
SRR13614388	OF_24	OF	Asia	China	116.65	37.3
SRR13614387	OF_25	OF	Asia	China	116.65	37.3
SRR13614386	OF_26	OF	Asia	China	116.65	37.3
SRR2071948	NOF_16	NOF	Asia	Vietnam	105.727278	21.08839
SRR2071949	OF_28	OF	Asia	Vietnam	105.727361	21.088395
SRR2070862	NOF_17	NOF	Asia	Vietnam	105.727278	21.08839
SRR2070865	OF_29	OF	Asia	Vietnam	105.727361	21.088395
ERR4878502	OF_40	OF	Europe	Germany	6.1375	51.7866
ERR4878503	OF_44	OF	Europe	Germany	6.1375	51.7866
ERR4878504	OF_46	OF	Europe	Germany	6.1375	51.7866

ERR4878505	OF_53	OF	Europe	Germany	6.1375	51.7866
ERR4878506	OF_54	OF	Europe	Germany	6.1375	51.7866
ERR4878507	OF_55	OF	Europe	Germany	6.1375	51.7866
ERR4878508	OF_56	OF	Europe	Germany	6.1375	51.7866
ERR4878509	OF_57	OF	Europe	Germany	6.1375	51.7866
ERR4878510	OF_58	OF	Europe	Germany	6.1375	51.7866
ERR4878511	OF_41	OF	Europe	Germany	6.1375	51.7866
ERR4878512	OF_42	OF	Europe	Germany	6.1375	51.7866
ERR4878513	OF_43	OF	Europe	Germany	6.1375	51.7866
ERR4245117	NOF_26	NOF	Europe	Germany	11.7	51.82
ERR4245118	NOF_27	NOF	Europe	Germany	11.7	51.82
ERR4245119	NOF_28	NOF	Europe	Germany	11.7	51.82
ERR4245120	NOF_29	NOF	Europe	Germany	11.7	51.82
ERR4245121	NOF_30	NOF	Europe	Germany	11.7	51.82
ERR4245122	NOF_31	NOF	Europe	Germany	11.7	51.82
ERR4245123	NOF_32	NOF	Europe	Germany	11.7	51.82
ERR4245124	NOF_33	NOF	Europe	Germany	11.7	51.82
ERR4245125	NOF_34	NOF	Europe	Germany	11.7	51.82
ERR4245126	NOF_35	NOF	Europe	Germany	11.7	51.82
ERR4245127	NOF_36	NOF	Europe	Germany	11.7	51.82
ERR4245128	NOF_37	NOF	Europe	Germany	11.7	51.82
ERR4245129	NOF_38	NOF	Europe	Germany	11.7	51.82
ERR4245130	NOF_39	NOF	Europe	Germany	11.7	51.82
ERR4245131	NOF_40	NOF	Europe	Germany	11.7	51.82
ERR4245132	NOF_41	NOF	Europe	Germany	11.7	51.82
SRR5259830	NOF_42	NOF	North America	The United States	-121.653	38.1087
SRR5259839	NOF_53	NOF	North America	The United States	-121.653	38.1087
SRR5259939	NOF_55	NOF	North America	The United States	-121.653	38.1087
SRR5690554	NOF_43	NOF	North America	The United States	-111.8143	41.7655
SRR12823096	NOF_44	NOF	North America	The United States	-119.74	46.25

SRR12823101	NOF_45	NOF	North America	The United States	-119.74	46.25
SRR12823104	NOF_46	NOF	North America	The United States	-119.74	46.25
SRR12823108	NOF_47	NOF	North America	The United States	-119.74	46.25
SRR12823112	NOF_48	NOF	North America	The United States	-119.74	46.25
SRR12823115	NOF_49	NOF	North America	The United States	-119.74	46.25
SRR12823118	NOF_50	NOF	North America	The United States	-119.74	46.25
SRR10113573	OF_102	OF	Asia	China	120.95024	30.5907243
SRR10113582	OF_103	OF	Asia	China	120.95024	30.5907243
SRR10113588	OF_104	OF	Asia	China	120.95024	30.5907243
SRR10767026	OF_105	OF	Asia	India	85.55	20.25
SRR10767027	OF_106	OF	Asia	India	85.55	20.25
SRR10767028	OF_107	OF	Asia	India	85.55	20.25
SRR12192804	NOF_111	NOF	Africa	South Africa	26.529	-25.9945
SRR12192805	NOF_112	NOF	Africa	South Africa	26.529	-25.9945
SRR12192851	NOF_113	NOF	Africa	South Africa	27.555	-26.19778
SRR12192852	NOF_114	NOF	Africa	South Africa	27.555	-26.19778
SRR12285196	NOF_115	NOF	Africa	South Africa	25.639	-25.8
SRR12285197	NOF_116	NOF	Africa	South Africa	25.639	-25.8
SRR12288316	NOF_117	NOF	Africa	South Africa	26.8886	-26.327
SRR12288317	NOF_118	NOF	Africa	South Africa	26.8886	-26.327
SRR12376371	NOF_124	NOF	Asia	India	76.16	27.53
SRR12875365	OF_109	OF	Asia	Indonesia	106.7435	-6.8286
SRR12875366	OF_110	OF	Asia	Indonesia	106.7435	-6.8286
SRR13484447	OF_111	OF	Asia	China	112.7	28.2
SRR13484448	OF_112	OF	Asia	China	112.7	28.2
SRR13484449	OF_113	OF	Asia	China	112.7	28.2
SRR13708140	OF_114	OF	Asia	China	117.08	36.11
SRR13708141	OF_115	OF	Asia	China	117.08	36.11
SRR13708142	OF_116	OF	Asia	China	117.08	36.11
SRR13708147	OF_117	OF	Asia	China	117.08	36.11

SRR13708148	OF_118	OF	Asia	China	117.08	36.11
SRR13870169	OF_120	OF	Asia	China	113	28.21
SRR13870180	OF_121	OF	Asia	China	113	28.21
SRR13870181	OF_122	OF	Asia	China	113	28.21
SRR14292182	OF_123	OF	Asia	China	104.06666	30.66667
SRR14292183	OF_124	OF	Asia	China	104.06666	30.66667
SRR14292190	OF_125	OF	Asia	China	106.71666	26.26666
SRR14292191	OF_126	OF	Asia	China	106.71666	26.26666
SRR14292192	OF_127	OF	Asia	China	106.71666	26.26666
SRR14292193	OF_128	OF	Asia	China	106.71666	26.26666
SRR14292194	OF_129	OF	Asia	China	104.06666	30.66667
SRR14292195	OF_131	OF	Asia	China	104.06666	30.66667
SRR14301315	OF_264	OF	North America	Canada	-81.2	43.01
SRR14301317	OF_265	OF	North America	Canada	-81.2	43.01
SRR14301318	OF_266	OF	North America	Canada	-81.2	43.01
SRR14320327	NOF_125	NOF	Asia	China	118.62	37.3
SRR14320330	NOF_126	NOF	Asia	China	118.62	37.3
SRR14320331	NOF_127	NOF	Asia	China	118.62	37.3
SRR14661182	NOF_128	NOF	Asia	China	115.17	36.78
SRR14661183	NOF_129	NOF	Asia	China	115.17	36.78
SRR14661184	NOF_130	NOF	Asia	China	115.17	36.78
SRR14661185	NOF_131	NOF	Asia	China	115.17	36.78
SRR14661186	NOF_132	NOF	Asia	China	115.17	36.78
SRR14661187	NOF_133	NOF	Asia	China	115.17	36.78
SRR14661188	NOF_134	NOF	Asia	China	115.17	36.78
SRR14661189	NOF_135	NOF	Asia	China	115.17	36.78
SRR14661190	NOF_136	NOF	Asia	China	115.17	36.78
SRR15168755	OF_267	OF	North America	The United States	-104.9995	40.6529
SRR15646751	OF_133	OF	Asia	China	100.41	25.38
SRR15646754	OF_134	OF	Asia	China	100.41	25.38

SRR15646755	OF_135	OF	Asia	China	100.41	25.38
SRR15646757	OF_136	OF	Asia	China	102.93	24.67
SRR15646758	OF_137	OF	Asia	China	102.93	24.67
SRR15646759	OF_138	OF	Asia	China	102.93	24.67
SRR15910693	OF_139	OF	Asia	China	113.585	28.9266
SRR15910694	OF_141	OF	Asia	China	113.585	28.9266
SRR15910699	OF_142	OF	Asia	China	113.585	28.9266
SRR15910700	OF_143	OF	Asia	China	113.585	28.9266
SRR16106684	OF_144	OF	Asia	China	109.76373	21.50715
SRR16106685	OF_145	OF	Asia	China	109.76373	21.50715
SRR16106686	OF_146	OF	Asia	China	109.76373	21.50715
SRR17629608	OF_147	OF	Asia	India	94.33675	27.203564
SRR17938969	OF_248	OF	Europe	Russian Federation	83.47	55.01
SRR19134800	OF_31	OF	Asia	China	91.924	28.949
SRR19134801	OF_32	OF	Asia	China	91.924	28.949
SRR19134802	OF_33	OF	Asia	China	91.924	28.949
SRR19134803	OF_34	OF	Asia	China	91.924	28.949
SRR19134804	OF_35	OF	Asia	China	91.924	28.949
SRR19134805	OF_37	OF	Asia	China	91.924	28.949
SRR21169165	NOF_137	NOF	Asia	China	117.05555	24.61777
SRR21169166	NOF_138	NOF	Asia	China	117.05555	24.61777
SRR21169167	NOF_139	NOF	Asia	China	117.05555	24.61777
SRR21169168	OF_149	OF	Asia	China	117.05555	24.61777
SRR21169169	OF_150	OF	Asia	China	117.05555	24.61777
SRR21169170	OF_151	OF	Asia	China	117.05555	24.61777
SRR21169171	OF_152	OF	Asia	China	117.05555	24.61777
SRR21169172	OF_153	OF	Asia	China	117.05555	24.61777
SRR21169173	OF_154	OF	Asia	China	117.05555	24.61777
SRR21617303	OF_155	OF	Asia	China	113.58	24.55
SRR21617304	OF_156	OF	Asia	China	113.57	24.55

SRR21617313	OF_157	OF	Asia	China	113.64	24.55
SRR21617314	OF_158	OF	Asia	China	113.63	24.55
SRR21617317	OF_159	OF	Asia	China	113.52	24.55
SRR21617318	OF_160	OF	Asia	China	113.52	24.55
ERR671910	NOF_100	NOF	Oceania	Australia	142.28934	-36.67146
ERR671911	NOF_105	NOF	Oceania	Australia	142.28934	-36.67146
ERR671912	NOF_106	NOF	Oceania	Australia	142.28934	-36.67146
ERR671913	NOF_107	NOF	Oceania	Australia	142.28934	-36.67146
ERR687897	NOF_108	NOF	Oceania	Australia	142.28934	-36.67146
ERR687898	NOF_109	NOF	Oceania	Australia	142.28934	-36.67146
ERR671918	OF_94	OF	Oceania	Australia	141.97633	-35.11641
ERR671919	OF_95	OF	Oceania	Australia	141.97633	-35.11641
ERR671920	OF_96	OF	Oceania	Australia	141.97633	-35.11641
ERR671921	OF_89	OF	Oceania	Australia	141.97633	-35.11641
ERR687889	OF_90	OF	Oceania	Australia	141.97633	-35.11641
ERR687890	OF_91	OF	Oceania	Australia	141.97633	-35.11641
ERR687891	OF_92	OF	Oceania	Australia	141.97633	-35.11641
ERR687892	OF_93	OF	Oceania	Australia	141.97633	-35.11641
SRR14301321	OF_59	OF	North America	Canada	-81.2	43.01
SRR14301320	OF_60	OF	North America	Canada	-81.2	43.01
SRR14301319	OF_61	OF	North America	Canada	-81.2	43.01
SRR14301300	OF_62	OF	North America	Canada	-81.2	43.01
SRR14301316	OF_63	OF	North America	Canada	-81.2	43.01
SRR14301308	OF_64	OF	North America	Canada	-81.2	43.01
SRR14301304	OF_65	OF	North America	Canada	-81.2	43.01
SRR14301299	OF_66	OF	North America	Canada	-81.2	43.01
SRR14301298	OF_67	OF	North America	Canada	-81.2	43.01
SRR14301324	OF_68	OF	North America	Canada	-81.2	43.01
SRR14301323	OF_69	OF	North America	Canada	-81.2	43.01
SRR14301322	OF_70	OF	North America	Canada	-81.2	43.01

SRR14301301	OF_71	OF	North America	Canada	-81.2	43.01
SRR14301307	OF_72	OF	North America	Canada	-81.2	43.01
SRR14301306	OF_73	OF	North America	Canada	-81.2	43.01
SRR14301305	OF_74	OF	North America	Canada	-81.2	43.01
SRR14301303	OF_75	OF	North America	Canada	-81.2	43.01
SRR14301302	OF_76	OF	North America	Canada	-81.2	43.01
SRR17938970	OF_45	OF	Europe	Russian Federation	83.47	55.01
SRR17938971	OF_47	OF	Europe	Russian Federation	83.47	55.01
SRR17938972	OF_48	OF	Europe	Russian Federation	83.47	55.01
SRR17938973	OF_49	OF	Europe	Russian Federation	83.47	55.01
SRR17938974	OF_50	OF	Europe	Russian Federation	83.47	55.01
SRR17938981	OF_51	OF	Europe	Russian Federation	83.47	55.01
SRR17938982	OF_52	OF	Europe	Russian Federation	83.47	55.01
SRR7013883	NOF_60	NOF	North America	Canada	-107.8	50.28
SRR7013888	NOF_61	NOF	North America	Canada	-107.8	50.28
SRR7013866	NOF_62	NOF	North America	Canada	-107.8	50.28
SRR7013886	NOF_63	NOF	North America	Canada	-107.8	50.28
SRR7013881	NOF_64	NOF	North America	Canada	-107.8	50.28
SRR7013905	NOF_65	NOF	North America	Canada	-107.8	50.28
SRR7013890	NOF_66	NOF	North America	Canada	-107.8	50.28
SRR7013880	NOF_67	NOF	North America	Canada	-107.8	50.28
SRR7013868	NOF_68	NOF	North America	Canada	-107.8	50.28
SRR7013897	NOF_69	NOF	North America	Canada	-107.8	50.28
SRR7013870	NOF_70	NOF	North America	Canada	-107.8	50.28
SRR7013902	NOF_71	NOF	North America	Canada	-107.8	50.28
SRR7013882	NOF_72	NOF	North America	Canada	-107.8	50.28
SRR7013904	NOF_73	NOF	North America	Canada	-107.8	50.28
SRR7013872	NOF_74	NOF	North America	Canada	-107.8	50.28
SRR7013858	NOF_75	NOF	North America	Canada	-107.8	50.28
SRR7013876	NOF_76	NOF	North America	Canada	-107.8	50.28

SRR7013864	NOF_77	NOF	North America	Canada	-107.8	50.28
SRR7013895	NOF_78	NOF	North America	Canada	-107.8	50.28
SRR7013861	NOF_79	NOF	North America	Canada	-107.8	50.28
SRR7013896	NOF_80	NOF	North America	Canada	-107.8	50.28
SRR7013877	NOF_81	NOF	North America	Canada	-107.8	50.28
SRR7013906	NOF_82	NOF	North America	Canada	-107.8	50.28
SRR7013907	NOF_83	NOF	North America	Canada	-107.8	50.28
SRR7013879	NOF_84	NOF	North America	Canada	-107.8	50.28
SRR7013860	NOF_85	NOF	North America	Canada	-107.8	50.28
SRR7013859	NOF_86	NOF	North America	Canada	-107.8	50.28
SRR7013863	NOF_87	NOF	North America	Canada	-107.8	50.28
SRR7013871	NOF_88	NOF	North America	Canada	-107.8	50.28
SRR7013891	NOF_89	NOF	North America	Canada	-107.8	50.28
SRR7013899	NOF_90	NOF	North America	Canada	-107.8	50.28
SRR7013898	NOF_91	NOF	North America	Canada	-107.8	50.28
SRR7013869	NOF_92	NOF	North America	Canada	-107.8	50.28
SRR7013875	NOF_93	NOF	North America	Canada	-107.8	50.28
SRR7013878	NOF_94	NOF	North America	Canada	-107.8	50.28
SRR7013887	NOF_95	NOF	North America	Canada	-107.8	50.28
SRR7013885	NOF_96	NOF	North America	Canada	-107.8	50.28
SRR7013884	NOF_97	NOF	North America	Canada	-107.8	50.28
SRR7013865	NOF_98	NOF	North America	Canada	-107.8	50.28
SRR7013892	NOF_99	NOF	North America	Canada	-107.8	50.28
SRR16123970	OF_269	OF	North America	The United States	-121.871302	38.540587
SRR16123974	OF_270	OF	North America	The United States	-121.870682	38.540611
SRR16123980	OF_271	OF	North America	The United States	-121.875957	38.545045
SRR16123993	OF_272	OF	North America	The United States	-121.874259	38.543123
SRR16123997	OF_273	OF	North America	The United States	-121.874896	38.543092
SRR16124003	OF_274	OF	North America	The United States	-121.876575	38.545011
SRR16123964	OF_77	OF	North America	The United States	-121.876549	38.544366

SRR16123976	OF_78	OF	North America	The United States	-121.871718	38.541886
SRR16123977	OF_79	OF	North America	The United States	-121.871718	38.541886
SRR16123979	OF_80	OF	North America	The United States	-121.871537	38.541894
SRR16123982	OF_81	OF	North America	The United States	-121.871537	38.541894
SRR16123984	OF_82	OF	North America	The United States	-121.875162	38.541763
SRR16123986	OF_83	OF	North America	The United States	-121.875162	38.541763
SRR16123988	OF_84	OF	North America	The United States	-121.874985	38.541811
SRR16123989	OF_85	OF	North America	The United States	-121.874985	38.541811
SRR16123999	OF_86	OF	North America	The United States	-121.876379	38.544363
SRR16124000	OF_87	OF	North America	The United States	-121.876379	38.544363
SRR16123962	OF_88	OF	North America	The United States	-121.876549	38.544366
ERR7672977	NOF_165	NOF	Europe	The United Kingdom	-1.605268	52.205322
ERR7672978	OF_249	OF	Europe	The United Kingdom	-1.605268	52.205322
ERR7672979	NOF_166	NOF	Europe	The United Kingdom	-1.605268	52.205322
ERR7672980	OF_251	OF	Europe	The United Kingdom	-1.605268	52.205322
ERR1939165	NOF_56	NOF	North America	The United States	-88.242	40.075
ERR1939167	NOF_57	NOF	North America	The United States	-88.242	40.075
ERR1939169	NOF_58	NOF	North America	The United States	-88.242	40.075
ERR1939171	NOF_59	NOF	North America	The United States	-88.242	40.075
SRR14458715	NOF_203	NOF	North America	The United States	-119.518373	36.597906
SRR14458716	NOF_204	NOF	North America	The United States	-119.518373	36.597906
SRR14458717	NOF_205	NOF	North America	The United States	-119.518373	36.597906
SRR14458718	NOF_206	NOF	North America	The United States	-119.518373	36.597906
SRR14458719	NOF_207	NOF	North America	The United States	-119.518373	36.597906
SRR14458720	NOF_209	NOF	North America	The United States	-119.518373	36.597906
SRR14458721	NOF_210	NOF	North America	The United States	-119.518373	36.597906
SRR14458723	NOF_211	NOF	North America	The United States	-119.518373	36.597906
SRR14458724	NOF_212	NOF	North America	The United States	-119.518373	36.597906
SRR14458725	NOF_213	NOF	North America	The United States	-119.518373	36.597906
SRR14458726	NOF_214	NOF	North America	The United States	-119.518373	36.597906

SRR14458727	NOF_215	NOF	North America	The United States	-119.518373	36.597906
SRR14458728	NOF_216	NOF	North America	The United States	-119.518373	36.597906
SRR14458729	OF_268	OF	North America	The United States	-119.518373	36.597906
SRR14458734	NOF_217	NOF	North America	The United States	-119.518373	36.597906
SRR14458737	NOF_219	NOF	North America	The United States	-119.518373	36.597906
SRR14458738	NOF_220	NOF	North America	The United States	-119.518373	36.597906
SRR14458732	NOF_221	NOF	North America	The United States	-119.518373	36.597906

Supplementary Table 2. **Importance ranking of genera for NOF and OF classification according to the Random Forest classification model.**

Genus	MeanDecreaseAccuracy	MeanDecreaseGini
Cytobacillus	5.066867544	1.415330112
Frigoriglobus	4.890445257	0.787378366
Botrimarina	4.836414521	1.014917978
Limnoglobus	4.595851102	1.043591914
Legionella	4.593898245	0.952147181
Humisphaera	4.505362369	0.885653464
Tardibacter	4.460508291	1.02608585
Erythrobacter	4.409716891	0.872883261
Paroceanicella	4.335528804	0.739380581
Pseudonocardia	4.314292146	1.017012886
Phycisphaera	4.240033749	0.761341249
Georhizobium	4.215222055	0.979211849
Geodermatophilus	4.119103953	0.791115613
Tsuneonella	4.015584372	0.546703437
Gemmata	3.950867919	0.607687137
Metabacillus	3.926153273	0.593344291
Porphyrobacter	3.886633534	0.549197982
Microlunatus	3.876847101	0.665632553
Chloracidobacterium	3.869053433	0.725044684
Rubrobacter	3.866363016	0.682881481
Parasphingopyxis	3.84075223	0.72604298
Actinomarinicola	3.721008816	0.450252244
Leptolyngbya	3.675330779	0.559725351
Methyloceanibacter	3.648938924	0.511003426
Mycolicibacterium	3.589843659	0.388344315
Rhodobacter	3.587719137	0.513085153
Flavisolibacter	3.520549325	0.21797956
Qipengyuania	3.506472244	0.71673119
Rhizorhabdus	3.502458935	0.347610667
Blastomonas	3.477613725	0.456274848
Croceicoccus	3.408799584	0.983806467
Telmatocola	3.404109363	0.469101031
Croceibacterium	3.391429194	0.278802985
Urbifossiella	3.382824052	0.268269968
Nakamurella	3.313687884	0.295764718
Sphingosinithalassobacter	3.273581755	0.609337339
Aquicella	3.271683731	0.386325427
Sediminicoccus	3.271163626	0.482490443
Methylocaldum	3.21895609	0.281245386

Roseovarius	3.199271239	0.451843016
Altererythrobacter	3.186750658	0.223087094
Thermomicrobium	3.176467714	0.359137368
Staphylococcus	3.156983045	0.464616084
Candidatus *Nitrosocosmicus*	3.134323148	0.275988023
Steroidobacter	3.128676296	0.146328974
Allokutzneria	3.125011024	0.47651838
Modestobacter	3.074776883	0.302662564
Brevibacillus	3.066305353	0.373649711
Saccharomonospora	3.004238425	0.33331072
Scytonema	2.978689367	0.36478388
Aminobacter	2.969158275	0.229182799
Elioraea	2.960996743	0.259780778
Rufibacter	2.945837218	0.185560682
Nitrosopumilus	2.938480107	0.177234025
Roseococcus	2.92952983	0.349751117
Skermanella	2.922373365	0.231787337
Miniimonas	2.866097742	0.187656363
Nitrosospira	2.844531789	0.13884895
Lysobacter	2.841708185	0.213187487
Rothia	2.787965094	0.376669159
Planctomyces	2.765937532	0.111583225
Salipiger	2.756197065	0.274220545
Streptomyces	2.74340013	0.229857676
Arenimonas	2.735066073	0.203465434
Arthrobacter	2.731617012	0.141671228
Virgibacillus	2.717773333	0.163720998
Micropruina	2.706452776	0.412212538
Pelagerythrobacter	2.691217533	0.389295883
Terricaulis	2.683362802	0.068775911
Indioceanicola	2.678795948	0.234517471
Chondromyces	2.658176877	0.268159765
Jiangella	2.655129817	0.142957907
Serratia	2.654944477	0.179125126
Paludibaculum	2.652048599	0.14245373
Luteimonas	2.649026446	0.150537885
Lacibacter	2.628624848	0.121730761
Fimbriimonas	2.618517614	0.108197901
Noviherbaspirillum	2.618395741	0.098981363
Pigmentiphaga	2.593828352	0.186087969
Rhodoferax	2.590115555	0.392259288
Citromicrobium	2.565023723	0.169183819
Catellatospora	2.547483378	0.138196427
Diaminobutyricimonas	2.537845859	0.112716147

Blastococcus	2.517531793	0.3299565
Auraticoccus	2.509051986	0.140726083
Pikeienuella	2.50636933	0.258899778
Roseomonas	2.506016547	0.18575959
Methylobacterium	2.505188022	0.220306642
Microvirga	2.499111863	0.085403529
Phyllobacterium	2.487522396	0.246789724
Gordonia	2.478773569	0.220444439
Plantibacter	2.473373587	0.177634543
Neobacillus	2.470665478	0.097363463
Miltoncostaea	2.465410131	0.08497456
Desulfobulbus	2.464876229	0.078698851
Methyloligella	2.434025189	0.100852735
Diaphorobacter	2.433714599	0.09791283
Iamia	2.423464043	0.189513376
Methanosarcina	2.421210423	0.152879328
Methylomicrobium	2.419886828	0.122554804
Aquihabitans	2.417577314	0.112477712
Hoeflea	2.389606619	0.199704427
Labrys	2.381986611	0.069373609
Sphingobacterium	2.372205523	0.175640383
Pelagibacterium	2.363599341	0.069204358
Anaeromyxobacter	2.354029031	0.175317908
Nocardia	2.344558502	0.071041382
Candidatus *Saccharimonas*	2.343947844	0.143911185
Celeribacter	2.336584082	0.074508421
Gemmatimonas	2.334210553	0.098195783
Pseudorhodoplanes	2.332999399	0.190118151
Thermobacillus	2.32723436	0.178639584
Rhodovulum	2.321129525	0.13625852
Intrasporangium	2.317532595	0.172815841
Methylococcus	2.307981981	0.071231734
Denitratisoma	2.299201648	0.151988311
Sphingosinicella	2.297637046	0.230885327
Komagataeibacter	2.290114978	0.194640305
Rhizobium	2.287677481	0.191115754
Egibacter	2.279939763	0.239490946
Deinococcus	2.278594227	0.137558221
Thermomonas	2.276050459	0.09589974
Novosphingopyxis	2.270894582	0.198443378
Geobacillus	2.244578038	0.270879281
Marinobacterium	2.239510899	0.224451717
Thermus	2.236041731	0.24233961
Thalassospira	2.235250796	0.095504445

Enterobacter	2.231044111	0.130176828
Sandaracinobacter	2.223921305	0.245870367
Youhaiella	2.220175915	0.073330935
Brevirhabdus	2.214006528	0.126139057
Sulfuricaulis	2.212941456	0.068824739
Aquibium	2.212016632	0.04481868
Bacillus	2.181914441	0.169864235
Rhodothermus	2.178878961	0.167483537
Actinotalea	2.17007412	0.033482969
Epidermidibacterium	2.167909112	0.103874162
Cobetia	2.165035021	0.228929069
Nonomuraea	2.160661841	0.054772474
Pseudocnuella	2.160151872	0.031400955
Nitrososphaera	2.157951642	0.154598968
Shewanella	2.152610658	0.224502363
Allosphingosinicella	2.146550575	0.107411994
Marinithermus	2.144393767	0.174932497
Symbiobacterium	2.140050792	0.175561281
Isosphaera	2.136075831	0.163074464
Usitatibacter	2.131061981	0.075807347
Gloeobacter	2.130842786	0.143214586
Geobacter	2.130616452	0.134777958
Thiodictyon	2.130434498	0.063675275
Mucisphaera	2.12945747	0.074739563
Azospirillum	2.125354357	0.216594467
Kaistia	2.12212946	0.139453264
Chloroflexus	2.12150208	0.204519095
Caulobacter	2.120468135	0.144398586
Vogesella	2.115028411	0.111882637
Halomarina	2.112035329	0.109499427
Spirosoma	2.105359383	0.077063554
Candidatus *Bipolaricaulis*	2.099597757	0.078266273
Melaminivora	2.093550187	0.106657087
Labilithrix	2.088820762	0.04859369
Cellulosimicrobium	2.082728511	0.070468496
Novosphingobium	2.076924829	0.103957656
Aeoliella	2.072720937	0.286318304
Niveispirillum	2.064920249	0.036417167
Singulisphaera	2.062459158	0.088245772
Paraconexibacter	2.057895173	0.106315417
Devosia	2.052534697	0.074305933
Stieleria	2.051905753	0.126904268
Mycolicibacter	2.049815514	0.117548634
Mesorhizobium	2.046090272	0.12767898

Haloterrigena	2.041053479	0.078902842
Archangium	2.040702293	0.093977661
Proteus	2.040371261	0.124007065
Tessaracoccus	2.036228077	0.201507202
Dactylosporangium	2.028925067	0.118579019
Anatilimnocola	2.025763701	0.072969363
Stella	2.024558157	0.034203044
Solidesulfovibrio	2.023755125	0.147167331
Sphingopyxis	2.022816434	0.160780348
Nitrosomonas	2.019416862	0.158333214
Sorangium	2.016124159	0.066444878
Brevilactibacter	2.013232934	0.068722615
Roseivivax	2.011789164	0.041442184
Thermococcus	2.008504009	0.127164851
Pulveribacter	2.007736877	0.032013309
Paludisphaera	1.988416421	0.03309013
Chryseoglobus	1.987173377	0.062739417
Roseiflexus	1.986631249	0.067066029
Pseudobacter	1.985252596	0.154758664
Propioniciclava	1.980922282	0.088971313
Polymorphum	1.980679013	0.19259483
Nitrosococcus	1.978142094	0.102966751
Pseudactinotalea	1.978094234	0.048539405
Polymorphobacter	1.975916717	0.086562921
Ralstonia	1.974129135	0.130810861
Rhodoplanes	1.970040172	0.100302063
Methylogaea	1.966060783	0.059622446
Hypericibacter	1.961130328	0.126068798
Cereibacter	1.961123164	0.155062435
Microterricola	1.957310165	0.066763633
Anaerolinea	1.953853331	0.106255503
Gimesia	1.953794733	0.124215828
Alicyclobacillus	1.952734475	0.112106237
Calothrix	1.941938236	0.096219066
Candidatus *Viadribacter*	1.941525914	0.156811817
Jeongeupia	1.94132814	0.031822195
Duganella	1.939672065	0.064958757
Phytohabitans	1.936789416	0.073475638
Desulfosarcina	1.929916369	0.082250602
Haliangium	1.929623334	0.072047822
Candidatus *Nitrotoga*	1.918857684	0.064532747
Caldilinea	1.915844442	0.057446283
Hyphomicrobium	1.910480321	0.035196622
Micrococcus	1.906293748	0.204453502

Lichenicola	1.906198803	0.055613284
Lichenihabitans	1.904491321	0.047696918
Phaeobacter	1.903485525	0.036580032
Clavibacter	1.899125272	0.099596331
Aureimonas	1.895842333	0.041564018
Synechococcus	1.894258119	0.084465393
Conexibacter	1.894033861	0.194207288
Pannonibacter	1.890678182	0.064414456
Rhizobacter	1.886258286	0.072289302
Yinghuangia	1.879994766	0.022449131
Nisaea	1.879133281	0.070728393
Cellvibrio	1.878822012	0.048996414
Sphingorhabdus	1.872371738	0.147865833
Ramlibacter	1.86925932	0.131554435
Cystobacter	1.865345863	0.074565384
Thauera	1.865014028	0.146301162
Paraurantiacibacter	1.863041063	0.111094425
Agrococcus	1.857227838	0.05184665
Acidipropionibacterium	1.854295316	0.034454577
Paralcaligenes	1.853748884	0.119084257
Filomicrobium	1.851404863	0.086836517
Streptococcus	1.849893401	0.094582449
Allochromatium	1.841753082	0.038480709
Xanthomonas	1.837203782	0.052724497
Luteipulveratus	1.832962967	0.059144517
Nitrospira	1.832903516	0.067120924
Halobaculum	1.831821994	0.036623559
Agrobacterium	1.829822542	0.048581141
Ereboglobus	1.828744104	0.160046376
Treponema	1.825898656	0.151164949
Simplicispira	1.823945608	0.055637321
Oceanithermus	1.823167637	0.023847102
Xylophilus	1.822231592	0.060337374
Salaquimonas	1.820631831	0.02450915
Luteitalea	1.817095575	0.066898971
Clostridium	1.811873985	0.042137025
Tautonia	1.810815954	0.093137944
Meiothermus	1.80971986	0.12296613
Variibacter	1.809359358	0.077936064
Herbaspirillum	1.800269617	0.067435755
Variovorax	1.793237326	0.027109356
Luteimicrobium	1.791378292	0.080349374
Thioalkalivibrio	1.791330994	0.118197204
Halorubrum	1.7911438	0.056241185

Methanoculleus	1.791035019	0.125651176
Acidothermus	1.789385653	0.115507049
Ruegeria	1.781032516	0.080894539
Pseudarthrobacter	1.773048204	0.066002377
Serpentinimonas	1.771871692	0.08677057
Isoptericola	1.769485003	0.059138796
Flavihumibacter	1.767674753	0.061345883
Ferribacterium	1.766333119	0.077786535
Gemmobacter	1.76157675	0.137378316
Agromyces	1.757015016	0.067743824
Methylorubrum	1.753527993	0.083626824
Actinoplanes	1.742552012	0.086999049
Dietzia	1.738927385	0.064139058
Pararhodospirillum	1.736408014	0.011261905
Pyxidicoccus	1.734708743	0.02092954
Acidovorax	1.734037314	0.031092407
Azoarcus	1.731719061	0.066981153
Kosakonia	1.730692602	0.035018984
Bdellovibrio	1.729077713	0.052451026
Luteolibacter	1.728171292	0.064841974
Thermobifida	1.726730459	0.083197436
Verminephrobacter	1.726321895	0.061482997
Aquabacterium	1.72613893	0.075074973
Spiractinospora	1.7255349	0.089434818
Cryobacterium	1.7232239	0.040931459
Maioricimonas	1.722890271	0.031378862
Rhodanobacter	1.720801714	0.053584523
Collinsella	1.720186204	0.044125044
Marisediminicola	1.71896457	0.053469042
Tumebacillus	1.715324229	0.157314027
Bradyrhizobium	1.707979826	0.116078118
Alienimonas	1.704600554	0.037256509
Cnuibacter	1.703623509	0.074971719
Saccharothrix	1.702585132	0.048357073
Leucobacter	1.69453049	0.052043881
Candidatus *Symbiobacter*	1.687519517	0.032815046
Oryzomicrobium	1.687376758	0.029186809
Polaromonas	1.68666936	0.068568913
Mycobacterium	1.68599605	0.112476125
Dermacoccus	1.684710906	0.036620294
Tomitella	1.682052277	0.020636508
Massilia	1.681578083	0.15999241
Mycolicibacillus	1.678218373	0.026131279
Microcella	1.676428949	0.045423132

Acetobacter	1.674017155	0.159121365
Olsenella	1.671039821	0.053563664
Shinella	1.670995319	0.054804876
Tepidimonas	1.669927267	0.017911308
Sandaracinus	1.668106844	0.036482836
Pontibacter	1.666687145	0.06354487
Achromobacter	1.664236583	0.029656533
Martelella	1.664115364	0.059244003
Granulicella	1.662126823	0.030612236
Starkeya	1.661792636	0.08503726
Thermomonospora	1.649509319	0.101951188
Paraoerskovia	1.64936784	0.075490298
Bythopirellula	1.647024004	0.032181796
Pelobacter	1.639577439	0.03222551
Acidisarcina	1.635741057	0.073697344
Pseudolysobacter	1.633875861	0.053694493
Bordetella	1.632410277	0.026095238
Phototrophicus	1.632319694	0.029558914
Pseudosulfitobacter	1.630149591	0.030700443
Ktedonosporobacter	1.629365222	0.028472727
Undibacterium	1.628071164	0.079154508
Salinibacterium	1.62760138	0.018130265
Citrifermentans	1.62541838	0.061471721
Sphaerotilus	1.619986848	0.05711865
Ancylobacter	1.61683864	0.040925977
Peribacillus	1.614946273	0.076548262
Bosea	1.614677685	0.045954936
Thioflavicoccus	1.613323226	0.078003978
Paenarthrobacter	1.6129802	0.060884533
Desulfarculus	1.61279146	0.045403764
Acidithiobacillus	1.610387171	0.089026463
Caulifigura	1.610058873	0.080728171
Paucibacter	1.607881237	0.045541524
Janthinobacterium	1.604887252	0.033219974
Ectothiorhodospira	1.604785734	0.03311413
Chthonomonas	1.604192198	0.047646039
Phycicoccus	1.595894426	0.027040855
Cupriavidus	1.595686532	0.08259166
Ferrigenium	1.593012571	0.124233053
Frigoribacterium	1.588213855	0.130977021
Persicimonas	1.588040669	0.023935007
Terriglobus	1.583796134	0.052061823
Pseudogulbenkiania	1.580668145	0.069323464
Sanguibacter	1.580576821	0.037618605

Ottowia	1.580065401	0.039302052
Gryllotalpicola	1.572805379	0.029794571
Azotobacter	1.571456873	0.088945404
Neisseria	1.570981255	0.073769294
Desulfomicrobium	1.555949312	0.04307518
Curtobacterium	1.55110576	0.082953536
Halorhodospira	1.540802785	0.13343771
Parolsenella	1.540773934	0.058037775
Immundisolibacter	1.535234144	0.035082159
Acidimicrobium	1.533820252	0.052377185
Frankia	1.527734242	0.075901781
Haematobacter	1.521886661	0.049432072
Chlorobaculum	1.520561491	0.039823877
Roseitalea	1.520091408	0.047479278
Hankyongella	1.517896535	0.016153257
Baekduia	1.513979777	0.04774087
Aquisphaera	1.510935522	0.133810713
Hyphomonas	1.508768664	0.023266071
Thioclava	1.508258437	0.043787688
Eggerthella	1.507896479	0.041679102
Ferrovibrio	1.505298819	0.036851495
Rosistilla	1.50076082	0.074031555
Selenomonas	1.495867774	0.08755556
Capnocytophaga	1.486011242	0.158540485
Dickeya	1.485812682	0.138479237
Sphingomonas	1.474194356	0.103468578
Prevotella	1.471500033	0.051249339
Tuwongella	1.457221504	0.093478842
Methylomagnum	1.454329345	0.023894574
Corynebacterium	1.445775024	0.080471026
Jatrophihabitans	1.442012986	0.086617453
Pelolinea	1.440181164	0.044087475
Sideroxydans	1.424106874	0.06058358
Nitrogeniibacter	1.42329841	0.040580161
Sinorhizobium	1.421648548	0.03344691
Burkholderia	1.417578666	0.032273848
Chryseolinea	1.417050503	0.038065354
Haloplanus	1.417050503	0.025078521
Granulibacter	1.417050503	0.014229963
Spiribacter	1.416994796	0.007580201
Lacunisphaera	1.416815398	0.059163103
Halovivax	1.416815398	0.018478095
Gephyromycinifex	1.416806612	0.029067268
Halopseudomonas	1.416806612	0.027164423

Edaphobacter	1.416806612	0.007466667
Symmachiella	1.416511938	0.018010256
Delftia	1.416491629	0.050490079
Phenylobacterium	1.416233777	0.026210826
Falsirhodobacter	1.416205397	0.013708244
Dinoroseobacter	1.416175512	0.02938271
Catenulispora	1.416175512	0.012707692
Alkalihalobacillus	1.416075693	0.038072593
Phnomibacter	1.415837373	0.011266443
Sagittula	1.415214853	0.00875174
Phreatobacter	1.415084048	0.027661594
Nocardiopsis	1.415084048	0.014121569
Mucilaginibacter	1.415084048	0.009841444
Aquabacter	1.414938758	0.010320582
Polynucleobacter	1.414938758	0.008728029
Defluviicoccus	1.414568388	0.026653594
Collimonas	1.414125725	0.036141683
Roseateles	1.41356023	0.018582888
Raineyella	1.413213502	0.020410928
Ahniella	1.413016187	0.09601982
Hoyosella	1.412859872	0.013340468
Silicimonas	1.412559373	0.006705835
Stappia	1.412136112	0.032870773
Aerosticca	1.411793262	0.00977381
Acidobacterium	1.410951664	0.106794892
Bremerella	1.406243202	0.021136296
Lacipirellula	1.406017395	0.068230192
Cohnella	1.40568924	0.048105001
Marmoricola	1.405410236	0.035695003
Tepidiforma	1.405100663	0.014709273
Gluconobacter	1.402384878	0.050027168
Dyadobacter	1.401907936	0.033901407
Psychrobacillus	1.400273435	0.134201798
Sterolibacterium	1.398135697	0.120753505
Pelagovum	1.397213773	0.039570215
Dokdonella	1.397110485	0.04011241
Salinibacter	1.392905865	0.034403485
Marichromatium	1.392032268	0.02753354
Tardiphaga	1.388742182	0.046080213
Dechloromonas	1.386975702	0.069409459
Salinisphaera	1.384393921	0.043886356
Asticcacaulis	1.380555181	0.042771229
Fulvivirga	1.379432432	0.031793222
Thiocapsa	1.37715085	0.034944538

Demequina	1.376466103	0.09550918
Yangia	1.374578563	0.044534244
Curvibacter	1.368040394	0.035753059
Marinobacter	1.36232155	0.057186061
Ideonella	1.360347783	0.012952381
Sporosarcina	1.357730837	0.105851358
Rhodospirillum	1.354050258	0.092876838
Sulfurifustis	1.353290406	0.015241918
Rhodopseudomonas	1.34740485	0.007820463
Quatrionicoccus	1.347228355	0.068260607
Corallococcus	1.347173593	0.014075817
Chitinolyticbacter	1.347120692	0.010603674
Nitrospirillum	1.34406228	0.027220482
Microbacterium	1.341949555	0.051525881
Aquisalimonas	1.340862477	0.020976492
Sideroxyarcus	1.339758218	0.028965821
Candidatus *Promineofilum*	1.339376819	0.10287474
Afipia	1.338805363	0.032211765
Tsukamurella	1.336245685	0.091713813
Paracoccus	1.334009152	0.063425466
Methylocella	1.331422542	0.050480226
Anoxybacillus	1.331196937	0.047556408
Mycetohabitans	1.330963147	0.01448505
Crenobacter	1.327466668	0.02190095
Melittangium	1.327069782	0.076560943
Amycolatopsis	1.326497378	0.023688889
Brevibacterium	1.323854186	0.109983912
Alistipes	1.319094732	0.047165271
Aeromicrobium	1.317051599	0.102991997
Desulfuromonas	1.316945747	0.069075778
Parvibaculum	1.316945747	0.018789651
Micromonospora	1.314286069	0.052201278
Runella	1.313391379	0.038628571
Niabella	1.313281884	0.038584141
Friedmanniella	1.307166499	0.02174733
Roseibium	1.305424598	0.020344912
Sulfuriferula	1.303663422	0.074058928
Mameliella	1.300530356	0.022401805
Alloacidobacterium	1.292697001	0.03543424
Georgenia	1.287190525	0.046308653
Bacteroides	1.287190525	0.042884738
Pirellulimonas	1.281088152	0.130677858
Actinomadura	1.27994566	0.015269846
Actinokineospora	1.276598073	0.007134426

Austwickia	1.275549417	0.033671874
Polaribacter	1.273423649	0.034287558
Blastochloris	1.270648817	0.021182429
Arachnia	1.270444292	0.010746032
Geomonas	1.262628285	0.052174444
Algoriphagus	1.259906152	0.018102857
Allosaccharopolyspora	1.256313447	0.044266651
Terrihabitans	1.254049344	0.067752481
Chryseobacterium	1.251782545	0.053215986
Ilumatobacter	1.249925817	0.049451879
Ruficoccus	1.248580611	0.041802377
Thiobacillus	1.247109264	0.073019499
Inhella	1.243241783	0.066634616
Gluconacetobacter	1.241264168	0.09810631
Sphaerobacter	1.234643622	0.070848628
Truepera	1.226783691	0.110705085
Roseobacter	1.225377559	0.058030075
Methylomonas	1.22110651	0.036421443
Desulfovibrio	1.220774308	0.043108295
Citrobacter	1.220016326	0.055060938
Stigmatella	1.217464725	0.058447582
Thermaerobacter	1.206958526	0.110945257
Niastella	1.195880425	0.019541667
Mycobacteroides	1.194067355	0.030750725
Nostoc	1.18892511	0.056307387
Protaetiibacter	1.18773909	0.120596886
Methylibium	1.186905304	0.02266372
Cellulomonas	1.185922851	0.09516558
Mycetocola	1.184517246	0.022293333
Actinopolymorpha	1.170420228	0.030914734
Oceanidesulfovibrio	1.164829447	0.018875277
Frateuria	1.162940684	0.016299766
Syntrophobacter	1.162639657	0.063281455
Aurantimonas	1.161992499	0.00841232
Pimelobacter	1.159691983	0.017574883
Salinicola	1.159073033	0.027870236
Pedococcus	1.157643033	0.046017648
Adhaeribacter	1.157192782	0.035916985
Polymorphospora	1.156622311	0.034971167
Pyruvatibacter	1.150033496	0.017514058
Adhaeretor	1.114119733	0.025068853
Xanthobacter	1.103984197	0.056308407
Segniliparus	1.102115207	0.0434062
Kaustia	1.100802143	0.015389831

Paenibacillus	1.098682065	0.044317728
Sulfuritortus	1.098482844	0.065507838
Occultella	1.088928225	0.047489647
Aromatoleum	1.064619593	0.035179832
Nordella	1.051760126	0.095132759
Roseimaritima	1.047217216	0.047972275
Rhodocytophaga	1.046355508	0.05035057
Leifsonia	1.045491109	0.077409281
Sphingobium	1.039276534	0.073414407
Oscillatoria	1.014463868	0.061951678
Roseimicrobium	1.014433201	0.109561858
Actinomyces	1.001001503	0.072453188
Sulfuritalea	1.001001503	0.05498131
Nibricoccus	1.001001503	0.051770936
Opitutus	1.001001503	0.049989947
Bifidobacterium	1.001001503	0.049542044
Edwardsiella	1.001001503	0.048710957
Acuticoccus	1.001001503	0.046322373
Brachybacterium	1.001001503	0.039655083
Kitasatospora	1.001001503	0.036936035
Kutzneria	1.001001503	0.034302875
Polyangium	1.001001503	0.033846422
Ornithinimicrobium	1.001001503	0.033432975
Orrella	1.001001503	0.03204127
Sulfitobacter	1.001001503	0.029312821
Comamonas	1.001001503	0.028723618
Rhodovastum	1.001001503	0.02848749
Halomonas	1.001001503	0.027370439
Citricoccus	1.001001503	0.027242424
Tetrasphaera	1.001001503	0.026320346
Schaalia	1.001001503	0.025967805
Actinopolyspora	1.001001503	0.024513043
Halobacterium	1.001001503	0.023789899
Sulfurivermis	1.001001503	0.023625397
Syntrophotalea	1.001001503	0.022956522
Nitrobacter	1.001001503	0.022841119
Exiguobacterium	1.001001503	0.021977408
Lignipirellula	1.001001503	0.021833117
Fibrella	1.001001503	0.021223817
Tabrizicola	1.001001503	0.020228448
Hyphobacterium	1.001001503	0.019455649
Herbiconiux	1.001001503	0.018658421
Frondihabitans	1.001001503	0.018609091
Caballeronia	1.001001503	0.018238095

Humibacter	1.001001503	0.017889474
Actinoalloteichus	1.001001503	0.01764
Panacibacter	1.001001503	0.017570691
Hydrogenibacillus	1.001001503	0.016806723
Pseudomonas	1.001001503	0.016
Actinosynnema	1.001001503	0.015652174
Castellaniella	1.001001503	0.015268376
Dehalogenimonas	1.001001503	0.01524097
Pandoraea	1.001001503	0.014540476
Verrucomicrobium	1.001001503	0.013592689
Hartmannibacter	1.001001503	0.0135
Pectobacterium	1.001001503	0.013333333
Aquisediminimonas	1.001001503	0.013212121
Halorussus	1.001001503	0.012467532
Thiomonas	1.001001503	0.011166276
Natronomonas	1.001001503	0.010804466
Filimonas	1.001001503	0.010689177
Erwinia	1.001001503	0.010616357
Frigidibacter	1.001001503	0.010178437
Paraburkholderia	1.001001503	0.010169697
Fuscovulum	1.001001503	0.00974026
Acidihalobacter	1.001001503	0.008927273
Verrucosispora	1.001001503	0.008213904
Haloprofundus	1.001001503	0.008126984
Ponticoccus	1.001001503	0.007822134
Salinispora	1.001001503	0.00734864
Myxococcus	1.001001503	0.007314286
Oleiharenicola	1.001001503	0.007272727
Acidiphilium	1.001001503	0.007254658
Candidatus *Koribacter*	1.001001503	0.007058824
Agreia	1.001001503	0.007
Natronosporangium	1.001001503	0.006878788
Schlegelella	1.001001503	0.006666667
Gemmatirosa	1.001001503	0.006612613
Pirellula	1.001001503	0.005940299
Acidiferrobacter	1.001001503	0.003935484
Kytococcus	1.001001503	0.003914894
Ferruginibacter	1.001001503	0.003789474
Neotabrizicola	1.001001503	0.003777778
Vulgatibacter	1.001001503	0.003756803
Leptothrix	1.001001503	0.003751773
Flagellatimonas	1.001001503	0.00374026
Vibrio	1.001001503	0.003666667
Alkalilimnicola	1.001001503	0.0036

Kineococcus	1.001001503	0.0032
Halosimplex	1.001001503	0.003
Methyloversatilis	1.001001503	0.002666667
Paludibacterium	1.001001503	0.002666667
Streptosporangium	1.001001503	0.001935484
Alcaligenes	1.001001503	0.001173333
Aeromonas	1.001001503	0.000609524
Couchioplanes	0.986369954	0.044935948
Rathayibacter	0.967997221	0.085100419
Cyanobium	0.959282214	0.062283057
Raoultella	0.953195867	0.100508869
Salmonella	0.943867551	0.03701356
Pseudolabrys	0.94349889	0.040010693
Rhodococcus	0.93593174	0.046938011
Glutamicibacter	0.931846756	0.05627241
Pseudoalteromonas	0.915674681	0.071994753
Niallia	0.902318224	0.107019479
Lentzea	0.857319242	0.06609926
Thiocystis	0.848311476	0.025841026
Kocuria	0.847330501	0.029142022
Alloactinosynnema	0.829038739	0.02977902
Kibdelosporangium	0.82171306	0.033015306
Thiohalobacter	0.817139863	0.064577584
Hylemonella	0.812056449	0.031355996
Neorhizobium	0.800712977	0.056142866
Labrenzia	0.797141636	0.017222816
Saccharibacillus	0.793183832	0.020731228
Chitinimonas	0.784947734	0.037973355
Cutibacterium	0.78082395	0.080972458
Solitalea	0.760940591	0.011612236
Limnohabitans	0.738194679	0.042546224
Methylosinus	0.707226768	0.029585193
Prauserella	0.68128777	0.04462864
Leclercia	0.643440934	0.018388094
Azospira	0.626040904	0.024439385
Sulfidibacter	0.616321145	0.059719825
Chitinophaga	0.600828586	0.045042931
Luteibacter	0.577733856	0.038450182
Paraflavitalea	0.536174862	0.018359544
Brevundimonas	0.510411603	0.070452739
Alicycliphilus	0.46498502	0.057781232
Serinicoccus	0.447303065	0.013
Solimonas	0.389623401	0.029951487
Mitsuaria	0.378975795	0.027421724

Niveibacterium	0.37779147	0.066116425
Actinocatenispora	0.376053754	0.010547726
Thermobispora	0.355538515	0.049500793
Klebsiella	0.318386358	0.021181406
Sinomonas	0.289921705	0.032185281
Escherichia	0.253526357	0.017488057
Wenzhouxiangella	0.231708268	0.008716667
Chelatococcus	0.166267862	0.0211679
Alcanivorax	0.136080775	0.07056645
Allobranchiibius	0.102582044	0.016907359
Methylocystis	0.097301772	0.029871403
Hymenobacter	0.025708742	0.075940767
Hydrogenophaga	0.012086856	0.039167411
Pengzhenrongella	0.012086856	0.028231461
Bradymonas	0	0.069484269
Microvirgula	0	0.059712028
Marinicauda	0	0.02725873
Stutzerimonas	0	0.023513043
Faecalibacterium	0	0.0232
Saccharopolyspora	0	0.020644855
Stenotrophomonas	0	0.016143247
Oricola	0	0.014861598
Skermania	0	0.01302381
Pedobacter	0	0.012885714
Subtercola	0	0.010784416
Mumia	0	0.010181818
Pseudodesulfovibrio	0	0.008545455
Aquitalea	0	0.007555556
Egicoccus	0	0.006545455
Cronobacter	0	0.006035714
Limnochorda	0	0.005333333
Chromobacterium	0	0.003777778
Plantactinospora	0	0.003671186
Acidibrevibacterium	0	0.003636364
Stackebrandtia	0	0.003555556
Pontivivens	0	0.0035
Yersinia	0	0.0034279
Flaviflexus	0	0.0032
Pukyongiella	0	0.003
Dyella	0	0.002937705
Janibacter	0	0.002666667
Pseudorhizobium	0	0.002666667
Ruania	0	0.002
Oerskovia	0	0.001827817

Brucella	0	0
Yimella	0	0
Nitratireductor	0	0
Methylacidimicrobium	0	0
Glaciihabitans	0	0
Leptospira	0	0
Nocardioides	0	0
Beutenbergia	0	0
Akkermansia	0	0
Pusillimonas	0	0
Methylovirgula	0	0
Vitreoscilla	0	0
Casimicrobium	0	0
Magnetospirillum	0	0
Propionibacterium	0	0
Microbulbifer	0	0
Leisingera	0	0
Lautropia	0	0
Kiritimatiella	0	0
Halotalea	0	0
Rhodomicrobium	0	0
Kribbella	0	0
Ensifer	0	0
Breoghania	-0.013216374	0.013333333
Arsenicicoccus	-0.038208092	0.011466667
Priestia	-0.046342855	0.019282407
Aquincola	-0.051392117	0.020990218
Lysinibacillus	-0.052697306	0.056772558
Kinneretia	-0.12146625	0.057352328
Tistrella	-0.158498849	0.030205025
Candidatus *Xiphinematobacter*	-0.24029854	0.045376852
Pseudoxanthomonas	-0.316984736	0.069951963
Sulfurimicrobium	-0.42594132	0.028749747
Acinetobacter	-0.438066394	0.011597576
Natrinema	-0.492863431	0.009328671
Streptacidiphilus	-0.566066968	0.008555556
Sulfuricella	-0.584462562	0.018765161
Gulosibacter	-0.786043763	0.018973372
Geotalea	-0.802403273	0.026171394
Rhodocaloribacter	-0.870245725	0.02655555
Sutterella	-0.883605848	0.051944849
Guyparkeria	-1.001001503	0.058890634
Rubrivivax	-1.001001503	0.020978738
Campylobacter	-1.001001503	0.0166

Planctopirus	-1.001001503	0.014985185
Paradevosia	-1.001001503	0.0145869
Ketogulonicigenium	-1.001001503	0.010737116
Xylanimonas	-1.001001503	0.009246422
Pantoea	-1.001001503	0.008564706
Ciceribacter	-1.001001503	0.007684706
Streptomonospora	-1.001001503	0.007662959
Arachidicoccus	-1.001001503	0.007644411
Beijerinckia	-1.001001503	0.007578198
Azorhizobium	-1.001001503	0.006933333
Aurantiacibacter	-1.001001503	0.004770833
Flavobacterium	-1.138741801	0.021191101
Caenibius	-1.172355223	0.043534658
Planococcus	-1.349551596	0.056045705
Euzebya	-1.737084657	0.012925253

Supplementary Table 3. **Detailed information of DESeq2 analysis.**

Genus	baseMean	log2FoldChange	lfcSE	stat	pvalue	padj	NOF	OF
Sphingobium	45149.66	0.3058	0.0824	3.7129	0.000204893	0.000559088	1.000%	1.065%
Massilia	34500.61	0.9118	0.1077	8.4672	2.51349E-17	3.84673E-16	0.578%	0.877%
Methylibium	4939.03	0.6060	0.0867	6.9890	2.76825E-12	2.47735E-11	0.096%	0.125%
Flavobacterium	13075.03	1.9187	0.1620	11.8464	2.24758E-32	1.31858E-30	0.152%	0.427%
Kocuria	4597.19	-0.5043	0.1360	-3.7078	0.000209045	0.000567483	0.130%	0.068%
Metabacillus	3111.81	-1.3106	0.2206	-5.9422	2.81273E-09	1.66867E-08	0.103%	0.036%
Rhodoplanes	11165.57	0.2448	0.0927	2.6394	0.008306367	0.015497391	0.256%	0.262%
Caulobacter	11825.98	0.5817	0.0827	7.0325	2.0283E-12	1.86251E-11	0.238%	0.287%
Aquincola	8017.30	0.6112	0.0843	7.2467	4.26898E-13	4.25287E-12	0.153%	0.202%
Aromatoleum	4958.29	0.4560	0.1013	4.5039	6.673E-06	2.35675E-05	0.102%	0.120%
Shinella	15384.78	0.3062	0.0732	4.1812	2.89996E-05	9.56987E-05	0.330%	0.397%
Aeromonas	18330.35	1.3437	0.1324	10.1486	3.36129E-24	9.85978E-23	0.248%	0.472%
Porphyrobacter	4717.25	-1.1332	0.1369	-8.2766	1.26692E-16	1.80793E-15	0.156%	0.064%
Paenibacillus	111453.68	0.4130	0.1612	2.5622	0.010400562	0.018871122	2.323%	2.501%
Azoarcus	6094.54	0.5629	0.1037	5.4254	5.78364E-08	2.80162E-07	0.117%	0.151%
Burkholderia	26465.24	0.2115	0.0919	2.3011	0.021386739	0.035848248	0.611%	0.554%
Microlunatus	4883.11	-0.7202	0.1090	-6.6087	3.87642E-11	3.00992E-10	0.150%	0.074%
Hydrogenophaga	49148.82	0.5220	0.0971	5.3748	7.66856E-08	3.64775E-07	0.967%	1.194%
Bosea	7018.12	0.2659	0.0704	3.7768	0.000158855	0.000443785	0.155%	0.152%
Brevundimonas	15208.72	1.2208	0.1135	10.7535	5.70807E-27	2.23249E-25	0.220%	0.413%
Microbacterium	35155.08	0.3029	0.0932	3.2505	0.00115219	0.002624842	0.736%	0.749%
Xanthomonas	19411.09	1.5039	0.1346	11.1744	5.4401E-29	2.49771E-27	0.237%	0.538%
Pandoraea	5312.91	0.3770	0.1000	3.7697	0.000163443	0.0004542	0.113%	0.124%

Frateuria	3194.21	0.6186	0.1569	3.9437	8.02219E-05	0.000242735	0.063%	0.114%
Microvirga	14465.57	-0.4920	0.1383	-3.5574	0.000374526	0.00095072	0.404%	0.234%
Roseomonas	9931.39	-0.1989	0.0830	-2.3947	0.016636058	0.028894207	0.256%	0.188%
Nonomuraea	9174.50	-0.3291	0.0969	-3.3971	0.000680979	0.001638072	0.245%	0.170%
Clostridium	21935.51	3.9038	0.2158	18.0917	3.70381E-73	3.91122E-70	0.060%	0.975%
Skermanella	35511.74	-0.4684	0.1387	-3.3766	0.000733782	0.001753108	0.966%	0.610%
Priestia	22139.12	0.8262	0.1461	5.6542	1.56552E-08	8.52161E-08	0.363%	0.559%
Azospirillum	5948.28	-0.3160	0.0673	-4.6936	2.68435E-06	9.94622E-06	0.164%	0.112%
Kutzneria	3976.10	-0.3869	0.1393	-2.7778	0.005472782	0.010702328	0.116%	0.069%
Phytohabitans	4140.73	-0.8702	0.1145	-7.5979	3.01089E-14	3.41882E-13	0.130%	0.059%
Arthrobacter	97940.02	1.2113	0.1251	9.6848	3.49757E-22	9.23359E-21	1.343%	2.489%
Tetrasphaera	3585.74	0.9865	0.1161	8.4992	1.90947E-17	2.96529E-16	0.057%	0.120%
Tessaracoccus	3686.68	-0.4852	0.0874	-5.5500	2.85633E-08	1.46422E-07	0.106%	0.063%
Sphaerotilus	18331.14	0.8222	0.1054	7.7975	6.31236E-15	7.66189E-14	0.315%	0.485%
Sphingomonas	87829.49	-0.4329	0.0711	-6.0925	1.1119E-09	7.07328E-09	2.581%	1.588%
Sinorhizobium	9716.76	-0.6251	0.1245	-5.0194	5.18265E-07	2.12952E-06	0.282%	0.139%
Brevibacterium	4456.89	-0.5699	0.1083	-5.2632	1.41558E-07	6.36105E-07	0.132%	0.067%
Planctomyces	5023.63	-0.1858	0.0762	-2.4372	0.014800924	0.02584372	0.132%	0.094%
Marinobacter	508.51	-0.2994	0.1189	-2.5172	0.01183014	0.021149226	0.111%	0.013%
Geodermatophilus	27510.81	0.3407	0.1700	2.0039	0.045075679	0.070894199	0.519%	0.504%
Gordonia	6743.81	-0.6970	0.0859	-8.1103	5.04764E-16	6.83373E-15	0.210%	0.102%
Acidovorax	39367.73	0.7695	0.0885	8.6966	3.42064E-18	5.82613E-17	0.696%	1.018%
Ramlibacter	8406.04	0.2221	0.0807	2.7527	0.005910592	0.01145245	0.189%	0.191%
Sinomonas	1473.65	0.8386	0.1713	4.8963	9.76582E-07	3.86244E-06	0.026%	0.160%
Saccharopolyspora	4816.71	0.2367	0.1146	2.0655	0.038875881	0.061919955	0.106%	0.096%
Rubrivivax	4402.09	0.9185	0.0980	9.3770	6.78482E-21	1.52442E-19	0.074%	0.117%

Tardibacter	3458.28	-1.1498	0.1280	-8.9822	2.65456E-19	5.00574E-18	0.123%	0.048%
Pedococcus	8585.26	1.1494	0.1324	8.6828	3.86058E-18	6.27195E-17	0.128%	0.211%
Castellaniella	3456.80	0.5161	0.1539	3.3532	0.00079897	0.001887499	0.066%	0.114%
Pseudonocardia	33453.56	-0.5876	0.0985	-5.9677	2.40688E-09	1.46917E-08	0.976%	0.536%
Enterobacter	18836.91	0.4815	0.1519	3.1697	0.001525827	0.003399311	0.385%	0.530%
Rubrobacter	7631.71	-0.8966	0.1679	-5.3385	9.37416E-08	4.38014E-07	0.219%	0.091%
Phycicoccus	8714.73	1.0224	0.1269	8.0545	7.97803E-16	1.0531E-14	0.133%	0.237%
Paenarthrobacter	8115.11	0.6955	0.1280	5.4334	5.53038E-08	2.71631E-07	0.144%	0.180%
Methylobacterium	13473.59	-0.2673	0.0854	-3.1311	0.001741525	0.003776817	0.364%	0.262%
Pedobacter	2263.09	1.6965	0.1815	9.3443	9.24831E-21	2.03463E-19	0.026%	0.120%
Rhodoferax	25462.70	0.4863	0.0945	5.1453	2.67034E-07	1.16044E-06	0.516%	0.639%
Mycolicibacterium	48592.65	0.4611	0.0719	6.4126	1.43025E-10	1.0069E-09	0.969%	1.149%
Anaeromyxobacter	26620.72	2.2065	0.1610	13.7040	9.60214E-43	1.26748E-40	0.206%	0.868%
Pannonibacter	3974.26	-0.2794	0.1409	-1.9834	0.047318265	0.073808106	0.101%	0.074%
Hypericibacter	4829.24	0.6983	0.1286	5.4303	5.62456E-08	2.73711E-07	0.086%	0.129%
Ideonella	7391.27	1.0026	0.1208	8.3020	1.02413E-16	1.48148E-15	0.118%	0.200%
Nocardiopsis	2675.91	0.3065	0.1318	2.3248	0.020082188	0.033930864	0.057%	0.174%
Pseudarthrobacter	24649.40	0.6912	0.1467	4.7109	2.46584E-06	9.26665E-06	0.424%	0.506%
Pelobacter	3387.15	1.8131	0.2325	7.7983	6.27241E-15	7.66189E-14	0.033%	0.101%
Iamia	4691.21	-0.2226	0.0945	-2.3562	0.018465455	0.031501648	0.119%	0.092%
Chryseobacterium	7391.22	1.8611	0.1618	11.4989	1.33665E-30	6.72142E-29	0.079%	0.243%
Pantoea	5025.65	1.4065	0.1877	7.4933	6.71678E-14	7.23767E-13	0.088%	0.414%
Actinoplanes	25716.51	-0.4707	0.1026	-4.5895	4.44337E-06	1.60692E-05	0.745%	0.432%
Epidermidibacterium	4683.41	-0.6229	0.1572	-3.9621	7.43082E-05	0.000228109	0.142%	0.077%
Stenotrophomonas	47820.47	1.2907	0.1101	11.7182	1.02781E-31	5.71249E-30	0.642%	1.264%
Gemmata	4659.15	-0.4620	0.0869	-5.3142	1.07151E-07	4.9628E-07	0.133%	0.076%

Delftia	19573.52	0.7224	0.0933	7.7448	9.57185E-15	1.1231E-13	0.352%	0.460%
Caballeronia	8166.58	-0.3535	0.1382	-2.5585	0.010512708	0.019041885	0.235%	0.156%
Agrobacterium	12199.97	1.7041	0.1407	12.1099	9.36117E-34	6.59027E-32	0.133%	0.375%
Phenylobacterium	13651.31	0.4202	0.0805	5.2176	1.8127E-07	8.0429E-07	0.289%	0.330%
Croceicoccus	5028.20	-0.5607	0.0979	-5.7258	1.02948E-08	5.69179E-08	0.151%	0.086%
Geobacter	4231.02	1.7232	0.1598	10.7843	4.08191E-27	1.7242E-25	0.043%	0.130%
Methylorubrum	4616.36	-0.2413	0.0779	-3.0974	0.001952198	0.00416469	0.120%	0.085%
Lysobacter	42313.51	0.3633	0.1111	3.2699	0.001075844	0.002459072	0.880%	0.959%
Pseudomonas	157997.61	0.9935	0.1038	9.5736	1.03226E-21	2.59645E-20	2.558%	3.803%
Devosia	8350.53	0.5774	0.1182	4.8855	1.03177E-06	4.06549E-06	0.143%	0.199%
Gemmatirosa	15979.79	-0.8511	0.1130	-7.5311	5.03218E-14	5.5354E-13	0.528%	0.241%
Stutzerimonas	6418.68	0.2572	0.1256	2.0471	0.040653109	0.064458984	0.146%	0.134%
Rhizorhabdus	13711.20	-0.5676	0.0728	-7.7982	6.28084E-15	7.66189E-14	0.422%	0.250%
Nocardia	10620.74	-0.2824	0.0793	-3.5600	0.000370903	0.000943791	0.285%	0.189%
Thauera	24445.50	0.4228	0.1226	3.4496	0.000561417	0.001378736	0.474%	0.580%
Erythrobacter	4423.17	-0.5465	0.0834	-6.5487	5.80323E-11	4.40878E-10	0.133%	0.075%
Sphingopyxis	19731.57	0.3293	0.1006	3.2728	0.001064741	0.002444276	0.423%	0.472%
Schlegelella	4665.87	0.3146	0.0813	3.8708	0.000108492	0.000319129	0.102%	0.109%
Cellulomonas	18810.77	-0.3748	0.0875	-4.2823	1.84948E-05	6.23978E-05	0.513%	0.322%
Pimelobacter	4338.60	-0.2397	0.0859	-2.7909	0.005255521	0.010296532	0.114%	0.084%
Peribacillus	5950.49	-0.3181	0.1550	-2.0519	0.040182127	0.063808009	0.146%	0.107%
Intrasporangium	4650.98	1.6474	0.1323	12.4498	1.40223E-35	1.13904E-33	0.052%	0.129%
Alicycliphilus	12868.80	0.3895	0.1067	3.6514	0.000260804	0.000686806	0.264%	0.304%
Archangium	11519.64	-0.3923	0.1055	-3.7188	0.000200141	0.000547537	0.314%	0.211%
Luteitalea	12936.70	-0.4206	0.0791	-5.3144	1.07027E-07	4.9628E-07	0.370%	0.244%
Enterococcus	1291.15	-0.7286	0.2702	-2.6961	0.007015291	0.013300084	0.116%	0.038%

Rhodanobacter	8253.21	1.2614	0.1625	7.7628	8.30574E-15	9.85489E-14	0.117%	0.384%
Bacillus	97284.91	0.8658	0.1329	6.5169	7.1762E-11	5.33666E-10	1.562%	2.314%
Pseudolabrys	11257.42	0.7262	0.1185	6.1282	8.88683E-10	5.7929E-09	0.207%	0.299%
Aerosticca	8915.19	0.5452	0.1716	3.1764	0.001491183	0.003329153	0.178%	0.223%
Cupriavidus	47104.94	0.2989	0.0895	3.3382	0.00084311	0.001969743	1.056%	1.163%
Corynebacterium	13986.61	0.3612	0.1302	2.7741	0.005536223	0.010786442	0.286%	0.290%
Azospira	7596.65	0.9930	0.1253	7.9277	2.2317E-15	2.90948E-14	0.118%	0.205%
Bradyrhizobium	221665.82	0.2821	0.0734	3.8439	0.000121109	0.00034943	4.983%	5.297%
Aureimonas	3592.14	-0.3495	0.0705	-4.9539	7.27449E-07	2.94324E-06	0.101%	0.068%
Luteimonas	5912.93	0.6267	0.1053	5.9514	2.65853E-09	1.59512E-08	0.121%	0.242%
Thermomonas	5961.61	0.7059	0.1133	6.2298	4.66978E-10	3.20214E-09	0.108%	0.146%
Couchioplanes	5809.01	-0.4893	0.1272	-3.8476	0.000119299	0.000345149	0.167%	0.094%
Denitratisoma	4456.29	0.5931	0.1467	4.0431	5.27566E-05	0.000165314	0.084%	0.111%
Kinneretia	4631.25	0.2925	0.0632	4.6307	3.64475E-06	1.32719E-05	0.103%	0.107%
Catellatospora	6848.84	-0.3191	0.1101	-2.8983	0.003751366	0.007502732	0.177%	0.121%
Jiangella	3828.67	-0.7132	0.1092	-6.5323	6.47629E-11	4.88497E-10	0.115%	0.057%
Rhizobacter	9373.16	1.0036	0.0932	10.7725	4.64184E-27	1.8853E-25	0.152%	0.258%
Nakamurella	4434.27	0.8634	0.1109	7.7850	6.97264E-15	8.36716E-14	0.077%	0.125%
Lentzea	16303.79	0.3911	0.1479	2.6443	0.008185298	0.015298539	0.334%	0.350%
Cellulosimicrobium	9875.42	0.4032	0.1368	2.9467	0.003212356	0.006561408	0.197%	0.244%
Rhodopseudomonas	5379.70	0.2511	0.0728	3.4487	0.000563193	0.001379887	0.123%	0.123%
Comamonas	37871.20	0.8449	0.1171	7.2158	5.36066E-13	5.24153E-12	0.644%	1.025%
Thiobacillus	5415.20	3.0047	0.2057	14.6051	2.60787E-48	9.17971E-46	0.032%	0.190%
Corallococcus	4443.94	-0.1372	0.0680	-2.0177	0.043623271	0.068858258	0.114%	0.089%
Mycobacterium	44795.37	0.6571	0.0783	8.3967	4.59271E-17	6.83085E-16	0.825%	1.159%

Supplementary Table 4. **Shared and specific ARG subtypes in NOF and OF.**

Uniq ARG subtypes of NOF	Shared ARG subtypes	Uniq ARG subtypes of OF
beta-lactam__blaZ	*aminoglycoside__aac(2')-I*	*aminoglycoside__aac(3)-IX*
beta-lactam__CARB-6	*aminoglycoside__aac(3)-I*	*aminoglycoside__aac(3)-VI*
beta-lactam__class D beta-lactamase	*aminoglycoside__aac(3)-II*	*aminoglycoside__ant(3'')-Ih-aac(6')-IId*
beta-lactam__CMY-100	*aminoglycoside__aac(3)-IIIa*	*aminoglycoside__aph(2'')-Ie*
beta-lactam__CTX-M-131	*aminoglycoside__aac(3)-IV*	*aminoglycoside__aph(2'')-IV*
beta-lactam__CTX-M-68	*aminoglycoside__aac(3)-VII*	*aminoglycoside__rmtB*
beta-lactam__IMP-27	*aminoglycoside__aac(3)-VIII*	*aminoglycoside__streptomycin_resistance_protein*
beta-lactam__IMP-4	*aminoglycoside__aac(3)-X*	*aminoglycoside__tunicamycin_resistance_protein*
beta-lactam__IND-15	*aminoglycoside__aac(6')-31*	*beta-lactam__ACT-13*
beta-lactam__IND-7	*aminoglycoside__aac(6')-I*	*beta-lactam__ACT-14*
beta-lactam__LRA-8	*aminoglycoside__aac(6')-II*	*beta-lactam__ACT-19*
beta-lactam__OKP-A	*aminoglycoside__aad(6)*	*beta-lactam__ACT-20*
beta-lactam__OXA-117	*aminoglycoside__aad(9)*	*beta-lactam__ACT-21*
beta-lactam__OXA-20	*aminoglycoside__aadA*	*beta-lactam__ACT-23*
beta-lactam__OXA-214	*aminoglycoside__aadB*	*beta-lactam__blaI*
beta-lactam__OXA-230	*aminoglycoside__aadD*	*beta-lactam__CARB-8*
beta-lactam__OXA-82	*aminoglycoside__aadE*	*beta-lactam__CfxA3*
beta-lactam__OXA-94	*aminoglycoside__aadK*	*beta-lactam__class B beta-lactamase*
beta-lactam__TEM-112	*aminoglycoside__ant(2'')-I*	*beta-lactam__CMY-40*
beta-lactam__TEM-120	*aminoglycoside__ant(4')-Ia*	*beta-lactam__CMY-44*
beta-lactam__TEM-125	*aminoglycoside__ant(9)-I*	*beta-lactam__CMY-61*
beta-lactam__TEM-127	*aminoglycoside__aph(2'')-II*	*beta-lactam__CMY-78*
beta-lactam__TEM-128	*aminoglycoside__aph(2'')-III*	*beta-lactam__CMY-9*
beta-lactam__TEM-130	*aminoglycoside__aph(3')-I*	*beta-lactam__CTX-M*
beta-lactam__TEM-136	*aminoglycoside__aph(3'')-I*	*beta-lactam__DHA-6*
beta-lactam__TEM-141	*aminoglycoside__aph(3')-IIb*	*beta-lactam__fmtC*

beta-lactam__TEM-145	aminoglycoside__aph(3''')-III	beta-lactam__FONA-6
beta-lactam__TEM-147	aminoglycoside__aph(4)-I	beta-lactam__FOX-1
beta-lactam__TEM-148	aminoglycoside__aph(6)-I	beta-lactam__FOX-5
beta-lactam__TEM-156	aminoglycoside__streptothricin_acetyltransferase	beta-lactam__FOX-9
beta-lactam__TEM-159	aminoglycoside__viomycin_phosphotransferase	beta-lactam__IMP-15
beta-lactam__TEM-162	bacitracin__bacA	beta-lactam__IMP-16
beta-lactam__TEM-166	bacitracin__bcrA	beta-lactam__IMP-22
beta-lactam__TEM-169	beta-lactam__ACT-16	beta-lactam__IMP-31
beta-lactam__TEM-182	beta-lactam__AER-1	beta-lactam__IMP-32
beta-lactam__TEM-183	beta-lactam__ampC	beta-lactam__IMP-44
beta-lactam__TEM-193	beta-lactam__blaR1	beta-lactam__IND-4
beta-lactam__TEM-208	beta-lactam__CARB-12	beta-lactam__LEN-1
beta-lactam__TEM-54	beta-lactam__CfxA2	beta-lactam__LEN-15
beta-lactam__TEM-67	beta-lactam__CGB-1	beta-lactam__LEN-17
beta-lactam__TEM-83	beta-lactam__class A beta-lactamase	beta-lactam__LEN-21
beta-lactam__TEM-90	beta-lactam__class C beta-lactamase	beta-lactam__LEN-5
beta-lactam__TEM-95	beta-lactam__CMY-1	beta-lactam__mecA
beta-lactam__TEM-96	beta-lactam__CMY-19	beta-lactam__mecR1
trimethoprim__dfrA22	beta-lactam__CMY-98	beta-lactam__MIR-2
unclassified__patB	beta-lactam__FEZ-1	beta-lactam__MOX-1
	beta-lactam__GOB-1	beta-lactam__MOX-5
	beta-lactam__IMP-11	beta-lactam__MOX-6
	beta-lactam__IMP-13	beta-lactam__MOX-7
	beta-lactam__IMP-33	beta-lactam__MUS-1
	beta-lactam__IMP-7	beta-lactam__OCH-8
	beta-lactam__IMP-8	beta-lactam__OXA-1
	beta-lactam__IND-1	beta-lactam__OXA-11
	beta-lactam__IND-14	beta-lactam__OXA-118

beta-lactam__IND-3
beta-lactam__IND-5
beta-lactam__IND-8
beta-lactam__IND-9
beta-lactam__JOHN-1
beta-lactam__KHM-1
beta-lactam__LRA-1
beta-lactam__LRA-12
beta-lactam__LRA-13
beta-lactam__LRA-17
beta-lactam__LRA-19
beta-lactam__LRA-2
beta-lactam__LRA-3
beta-lactam__LRA-5
beta-lactam__LRA-9
beta-lactam__mecI
beta-lactam__metallo-beta-lactamase
beta-lactam__MOX-4
beta-lactam__OXA-10
beta-lactam__OXA-114
beta-lactam__OXA-129
beta-lactam__OXA-205
beta-lactam__OXA-209
beta-lactam__OXA-211
beta-lactam__OXA-22
beta-lactam__OXA-236
beta-lactam__OXA-278
beta-lactam__OXA-29
beta-lactam__OXA-333
beta-lactam__OXA-334
beta-lactam__OXA-119
beta-lactam__OXA-12
beta-lactam__OXA-142
beta-lactam__OXA-145
beta-lactam__OXA-161
beta-lactam__OXA-18
beta-lactam__OXA-2
beta-lactam__OXA-21
beta-lactam__OXA-212
beta-lactam__OXA-213
beta-lactam__OXA-237
beta-lactam__OXA-243
beta-lactam__OXA-258
beta-lactam__OXA-3
beta-lactam__OXA-309
beta-lactam__OXA-324
beta-lactam__OXA-325
beta-lactam__OXA-327
beta-lactam__OXA-349
beta-lactam__OXA-350
beta-lactam__OXA-352
beta-lactam__OXA-354
beta-lactam__OXA-358
beta-lactam__OXA-359
beta-lactam__OXA-361
beta-lactam__OXA-4
beta-lactam__OXA-56
beta-lactam__OXA-62
beta-lactam__OXA-7
beta-lactam__OXA-96

beta-lactam__OXA-335	*beta-lactam__OXY-1*
beta-lactam__OXA-34	*beta-lactam__OXY-2*
beta-lactam__OXA-36	*beta-lactam__OXY-5*
beta-lactam__OXA-5	*beta-lactam__PER-2*
beta-lactam__OXA-50	*beta-lactam__PER-3*
beta-lactam__OXA-53	*beta-lactam__RTG-4*
beta-lactam__OXA-58	*beta-lactam__SHV*
beta-lactam__OXA-60	*beta-lactam__SHV-105*
beta-lactam__OXA-9	*beta-lactam__SHV-112*
beta-lactam__PBP-1A	*beta-lactam__SHV-12*
beta-lactam__PBP-1B	*beta-lactam__SHV-39*
beta-lactam__penA	*beta-lactam__SHV-6*
beta-lactam__PSE-1	*beta-lactam__TEM-186*
beta-lactam__SHV-1	*beta-lactam__VEB-1*
beta-lactam__SHV-53	*bleomycin__bleomycin_resistance_protein*
beta-lactam__SMB-1	*chloramphenicol__catD*
beta-lactam__TEM-1	*chloramphenicol__catS*
beta-lactam__TEM-102	*chloramphenicol__chloramphenicol and florfenicol resistance gene*
beta-lactam__TEM-117	*chloramphenicol__chloramphenicol_and_florfenicol_exporter*
beta-lactam__TEM-124	*MLS__lmrP*
beta-lactam__TEM-157	*MLS__msrC*
beta-lactam__TEM-171	*MLS__vatE*
beta-lactam__TEM-178	*MLS__vgbA*
beta-lactam__TEM-187	*multidrug__mtrE*
beta-lactam__TEM-197	*multidrug__qacA*
beta-lactam__TEM-30	*multidrug__qacG*
beta-lactam__TEM-89	*polymyxin__mcr-1.5*
beta-lactam__THIN-B	*polymyxin__mcr-3*

bleomycin__bleO
carbomycin__carA
chloramphenicol__cat
chloramphenicol__catA
chloramphenicol__catB
chloramphenicol__catQ
chloramphenicol__cmlA
chloramphenicol__cmrA
chloramphenicol__floR
fosfomycin__fosA
fosfomycin__fosB
fosfomycin__fosX
fosmidomycin__rosA
fosmidomycin__rosB
fusidic-acid__fusH
kasugamycin__ksgA
MLS__ereA
MLS__erm(31)
MLS__erm(33)
MLS__erm(35)
MLS__erm(36)
MLS__erm(38)
MLS__erm(39)
MLS__erm(TR)
MLS__ermA
MLS__ermB
MLS__ermC
MLS__ermE
MLS__ermF
puromycin__puromycin_resistance_protein
quinolone__qnrB
quinolone__qnrS
spectinomycin__spcN
sulfonamide__sul3
tetracycline__tet34
tetracycline__tetD
tetracycline__tetE
tetracycline__tetJ
trimethoprim__dfrA12
trimethoprim__dfrA13
trimethoprim__dfrA16
trimethoprim__dfrA17
unclassified__16S_rRNA_methylase
unclassified__antibiotic resistance rRNA adenine methyltransferase
unclassified__EvgA
unclassified__tmrB
vancomycin__vanC
vancomycin__vanT

MLS__ermG
MLS__ermO
MLS__ermT
MLS__ermX
MLS__lmrA
MLS__lmrB
MLS__lnuA
MLS__lnuB
MLS__lsa
MLS__macA
MLS__macB
MLS__mefA
MLS__mgtA
MLS__mphA
MLS__mphB
MLS__mphC
MLS__msrA
MLS__oleB
MLS__oleD
MLS__srmB
MLS__tlcC
MLS__vatA
MLS__vatB
MLS__vatG
MLS__vgaA
MLS__vgaD
MLS__vgaE
multidrug__abeS
multidrug__acrA
multidrug__acrB

multidrug__adeA
multidrug__adeB
multidrug__adeC
multidrug__adeJ
multidrug__adeK
multidrug__amrB
multidrug__bcr
multidrug__bpeE
multidrug__bpeF
multidrug__ceoB
multidrug__cmeB
multidrug__emrA
multidrug__emrB
multidrug__EmrB-QacA
multidrug__emrD
multidrug__emrE
multidrug__emrK
multidrug__major_facilitator_superfamily_transporter
multidrug__mdfA
multidrug__mdtA
multidrug__mdtB
multidrug__mdtC
multidrug__mdtD
multidrug__mdtE
multidrug__mdtF
multidrug__mdtG
multidrug__mdtH
multidrug__mdtK
multidrug__mdtL
multidrug__mdtM

multidrug__mdtN
multidrug__mdtO
multidrug__mdtP
multidrug__mepA
multidrug__mexA
multidrug__mexB
multidrug__mexC
multidrug__mexD
multidrug__mexE
multidrug__mexF
multidrug__mexG
multidrug__mexI
multidrug__mexT
multidrug__mexW
multidrug__mexY
multidrug__multidrug_ABC_transporter
multidrug__multidrug_transporter
multidrug__norA
multidrug__opcM
multidrug__opmD
multidrug__oprA
multidrug__oprC
multidrug__oprJ
multidrug__oprM
multidrug__oprN
multidrug__pmrA
multidrug__qacB
multidrug__qacEdelta1
multidrug__sdeY
multidrug__smeB

multidrug__smeC
multidrug__smeD
multidrug__smeE
multidrug__smeF
multidrug__TolC
multidrug__ykkC
multidrug__ykkD
polymyxin__arnA
polymyxin__icr-Mo
polymyxin__mcr-5
quinolone__abaQ
quinolone__mfpA
quinolone__norB
quinolone__qepA
rifamycin__arr
rifamycin__rifampin_monooxygenase
sulfonamide__sul1
sulfonamide__sul2
sulfonamide__sul4
tetracenomycin_C__tcmA
tetracycline__otrA
tetracycline__tcr3
tetracycline__tet31
tetracycline__tet32
tetracycline__tet35
tetracycline__tet36
tetracycline__tet39
tetracycline__tet40
tetracycline__tet41
tetracycline__tet43

tetracycline__tet44
tetracycline__tetA
tetracycline__tetB
tetracycline__tetC
tetracycline__tetG
tetracycline__tetH
tetracycline__tetK
tetracycline__tetL
tetracycline__tetM
tetracycline__tetO
tetracycline__tetP
tetracycline__tetQ
tetracycline__tetR
tetracycline__tetracycline_resistance_protein
tetracycline__tetS
tetracycline__tetT
tetracycline__tetV
tetracycline__tetW
tetracycline__tetX
tetracycline__tetX1
tetracycline__tetX2
tetracycline__tetX3
tetracycline__tetX4
tetracycline__tetX5
tetracycline__tetY
tetracycline__tetZ
trimethoprim__dfrA1
trimethoprim__dfrA14
trimethoprim__dfrA15
trimethoprim__dfrA20

trimethoprim__dfrA5
trimethoprim__dfrB1
trimethoprim__dfrB2
trimethoprim__dfrB3
trimethoprim__dfrB6
unclassified__ArlR
unclassified__cAMP-regulatory_protein
unclassified__cob(I)alamin_adenolsyltransferase
unclassified__cpxR
unclassified__EvgA
unclassified__gadX
unclassified__H-NS
unclassified__LuxR
unclassified__rpsD
unclassified__sdiA
unclassified__tsnR
vancomycin__vanA
vancomycin__vanB
vancomycin__vanD
vancomycin__vanE
vancomycin__vanG
vancomycin__vanH
vancomycin__vanM
vancomycin__vanR
vancomycin__vanS
vancomycin__vanW
vancomycin__vanX
vancomycin__vanY
vancomycin__vanZ

Supplementary Table 5. **Shared and specific MRG subtypes in NOF and OF.**

Uniq MRG subtypes of NOF	Shared MRG subtypes	Uniq MRG subtypes of OF
Copper(Cu)__CopH	*Mercury(Hg)__merD*	*Lead(Pb)__pbrD*
multimetal__cnrH	*multimetal__fecE*	*Copper(Cu)__cueP*
Iron(Fe)__pmrB	*multimetal__golT*	*Mercury(Hg)__merB1*
Zinc(Zn)__zraP	*Arsenic(As)__aioA*	*Cadmium(Cd)__frnE*
Copper(Cu)__copV	*multimetal__kmtR*	*Tungsten(W)__tupB*
	multimetal__dsbA	*multimetal__fptA*
	Copper(Cu)__corR	*Mercury(Hg)__merB2*
	Copper(Cu)__pcoD	*multimetal__nccB*
	Gold(Au)__gesB	*Lead(Pb)__pbrB/pbrC*
	Zinc(Zn)__soxS	*Mercury(Hg)__merH*
	Zinc(Zn)__znuB/yebI	*Mercury(Hg)__merB3*
	multimetal__fieF/yiip	*multimetal__irlS*
	multimetal__yfeC	*multimetal__trgB*
	Arsenic(As)__arsC	*Nickel(Ni)__nirD*
	Selenium(Se)__sodB	*multimetal__fbpB*
	Copper(Cu)__mmco	*Tellurium(Te)__terW*
	Nickel(Ni)__nirC	*Silver(Ag)__silE*
	Chromium(Cr)__srpC	*multimetal__cnrR/cnrX*
	multimetal__troB	*Iron(Fe)__pmrC*
	Tellurium(Te)__tehB	*Copper(Cu)__ycnK*
	Copper(Cu)__cuiD	*Tellurium(Te)__terZ*
	Copper(Cu)__cutE/lnt	*multimetal__fecD*
	Manganese(Mn)__mntP/yebN	*Arsenic(As)__aioE*

multimetal__modE
multimetal__dmeF
Tellurium(Te)__terE
Nickel(Ni)__nikC
Zinc(Zn)__mdtB
Chromium(Cr)__chrC
multimetal__zupT/ygiE
Mercury(Hg)__merP
Copper(Cu)__CopC
multimetal__czcA
Cadmium(Cd)__yodD
multimetal__actA
Copper(Cu)__cusS
multimetal__cnrA
Arsenic(As)__pstB
Copper(Cu)__copS
multimetal__ctpC
Nickel(Ni)__nikR
Arsenic(As)__aioX/aoxX
multimetal__nmtR
Chromium(Cr)__chrR
Copper(Cu)__CusC
multimetal__cusC/ylcB
multimetal__czcD
Iron(Fe)__pmrA
Iron(Fe)__acn
Zinc(Zn)__zneB/hmxB
Copper(Cu)__copY/tcrY
Chromium(Cr)__chrI
Zinc(Zn)__smtB/ziaR
Tungsten(W)__tupC
multimetal__nccH
multimetal__czrB
Nickel(Ni)__hupE
Gold(Au)__gesC
multimetal__smtA
multimetal__pgpA/ltpgpA
Copper(Cu)__copZ
multimetal__nccC
Tellurium(Te)__trgA
Copper(Cu)__tcrZ
multimetal__cznA

Tellurium(Te)__klaB/telA/kilB
Arsenic(As)__arsR
Lead(Pb)__pbrA
multimetal__mgtA
Arsenic(As)__arsR4
Arsenic(As)__arsM
multimetal__pitA
Copper(Cu)__csoR
Iron(Fe)__dpr/dps
multimetal__modB
Zinc(Zn)__zitB/ybgR
Iron(Fe)__fetA/ybbL
Nickel(Ni)__nrsS
multimetal__corC
Arsenic(As)__arsH
multimetal__cueA
Copper(Cu)__pcoA
Copper(Cu)__Cop_restriction_enzyme
multimetal__rcnB/yohN
Copper(Cu)__cutA
multimetal__sitB
Copper(Cu)__actP
multimetal__czcP
Mercury(Hg)__merA
Copper(Cu)__copL
multimetal__nrsD/nreB

Silver(Ag)__silS
Copper(Cu)__yfmP
Tellurium(Te)__terB
Arsenic(As)__acr3
Copper(Cu)__CopF
Iron(Fe)__ybtP
Chromium(Cr)__chrA
Nickel(Ni)__nikA
Nickel(Ni)__hoxN
Copper(Cu)__copB
Arsenic(As)__pstS
Cadmium(Cd)__yjaA
multimetal__ctpD
Copper(Cu)__tcrY
multimetal__acrD
Copper(Cu)__copG
Copper(Cu)__CopA
multimetal__czcC
Copper(Cu)__ctpV
Nickel(Ni)__nirA
Lead(Pb)__pbrR
Iron(Fe)__bfrA
Aluminium(Al)__ALU1-P
multimetal__czrA
multimetal__arsA
multimetal__yfeA

Iron(Fe)__dpsA
Copper(Cu)__cueR
multimetal__corD
Copper(Cu)__dsbC
multimetal__nia
Chromium(Cr)__oscA
Nickel(Ni)__hupE2
Copper(Cu)__pcoR
multimetal__zinT/yodA
Mercury(Hg)__merR
Copper(Cu)__CueO
multimetal__nczA
Copper(Cu)__tcrB
Silver(Ag)__silB
Arsenic(As)__arrB
Cadmium(Cd)__ygiW
Nickel(Ni)__ncrB
multimetal__corA
multimetal__perO
Arsenic(As)__arsP
Copper(Cu)__cutC
Chromium(Cr)__chrB1
Silver(Ag)__silF
Copper(Cu)__pcoC
Arsenic(As)__arsD
multimetal__yfeD

Arsenic(As)__aioR/aoxR
Copper(Cu)__comR/ycfQ
Nickel(Ni)__nikD
Copper(Cu)__CusA
Iron(Fe)__furA
multimetal__baeS
Copper(Cu)__cusB
multimetal__actR
multimetal__cmtR
Copper(Cu)__ycnJ
Nickel(Ni)__ncrY
multimetal__czcR
Copper(Cu)__CopD
Gold(Au)__golS
multimetal__zipB
Tellurium(Te)__terD
Arsenic(As)__arsR1
multimetal__rcnR/yohL
multimetal__mrdH
Silver(Ag)__silC
Aluminium(Al)__G2alt
multimetal__glpF
Vanadium(V)__mexI
multimetal__zntA/yhhO
Copper(Cu)__hmrR
multimetal__sitD

Arsenic(As)__arsC2
multimetal__cadC
Iron(Fe)__ideR
Copper(Cu)__corS
Mercury(Hg)__merF
multimetal__dsbB
Tellurium(Te)__tehA
Zinc(Zn)__zntR/yhdM
Copper(Cu)__cueR/ybbI
multimetal__robA
multimetal__troA
Copper(Cu)__pcoE
Nickel(Ni)__hupN
Selenium(Se)__sodA
Chromium(Cr)__ruvB
multimetal__nreB
multimetal__mntH/yfeP
multimetal__czcB
Cadmium(Cd)__ychH
Zinc(Zn)__zraR/hydH
Cadmium(Cd)__yhcN
Mercury(Hg)__merE
multimetal__cmeB
Zinc(Zn)__mdtA
Tungsten(W)__tupA
Cobalt(Co)__nhlF

Copper(Cu)__copM
Mercury(Hg)__merT-P
Copper(Cu)__Cop-unnamed
Copper(Cu)__Cop_inner_membrane
Mercury(Hg)__merT
Copper(Cu)__copR
multimetal__mreA
Arsenic(As)__pstA
multimetal__fbpC
Copper(Cu)__cinA
Copper(Cu)__CopB-like
Chromium(Cr)__nfsA
Zinc(Zn)__ziaA
multimetal__rcnA/yohM
multimetal__sitA
Arsenic(As)__arsR2
Cadmium(Cd)__cadR
multimetal__cusF/cusX
Arsenic(As)__arsI
Copper(Cu)__pcoB
multimetal__yieF
Nickel(Ni)__nrsR
multimetal__nccA
multimetal__modA
Lead(Pb)__pbrT
Mercury(Hg)__merG

multimetal__cadX
Cobalt(Co)__corT/coaT
Nickel(Ni)__ncrC
Arsenic(As)__arsC1
Silver(Ag)__silR
Mercury(Hg)__merB
Copper(Cu)__mctB
multimetal__cnrT
multimetal__czcE
Copper(Cu)__cutF/nlpE
Mercury(Hg)__merR2
multimetal__mntR
Zinc(Zn)__mdtC
Nickel(Ni)__nikB
Arsenic(As)__arsR3
Chromium(Cr)__chrB
Arsenic(As)__aioS/aoxS
Arsenic(As)__arsB
multimetal__yfeB
Copper(Cu)__bhsA/ycfR/comC
multimetal__dmeR
Copper(Cu)__copJ
Copper(Cu)__CusF
Zinc(Zn)__ziaR
Copper(Cu)__Cop_response_transcription_regulator
Copper(Cu)__pcoS

multimetal__yqjH
Chromium(Cr)__recG
Arsenic(As)__arsT
Nickel(Ni)__nrsA
Copper(Cu)__tcrA
Silver(Ag)__silA
multimetal__cusR/ylcA
multimetal__irlR
Copper(Cu)__cutO
multimetal__cusA/ybdE
multimetal__corR/coaR
Nickel(Ni)__nikE
Zinc(Zn)__znuC/yebM
Copper(Cu)__Cop_sensor_histidine_kinase
Iron(Fe)__ybtQ
Nickel(Ni)__ncrA
Tellurium(Te)__terC
Arsenic(As)__arrA
Arsenic(As)__aioA/aoxB
Chromium(Cr)__chrA1
multimetal__sitC
multimetal__cadD
multimetal__fpvA
multimetal__modC
Zinc(Zn)__zur/yjbK
multimetal__corB

Mercury(Hg)__merR1
multimetal__zraS/hydG
Zinc(Zn)__znuA/yebL
Copper(Cu)__yfmO
Chromium(Cr)__chrF
Copper(Cu)__ricR
Copper(Cu)__ctpG
multimetal__czcS
Iron(Fe)__fetB/ybbM
multimetal__actS
Arsenic(As)__pstC
multimetal__baeR
multimetal__mntA/ytgA
Arsenic(As)__arsC3
Silver(Ag)__silP

Supplementary Table 6. **The taxonomic novelty of the MAGs.**

MAGs ID	Domain	Phylum	Class	Order	Family	Genus	Species	Subspecies	Dataset
OF_MAG90	0.97	0.886	0.753	0.42	0.151	0.099	0.000171	0	0
OF_MAG62	0.861	0.476	0.0597	0.0392	0.00938	0.0152	5.72E-05	0	0
OF_MAG61	0.968	0.88	0.739	0.392	0.143	0.0871	0.000171	0	0
NOF_MAG24	0.991	0.967	0.928	0.753	0.515	0.265	0.000457	0	0
NOF_MAG18	0.933	0.748	0.455	0.141	0.0294	0.0221	0.000171	0	0
NOF_MAG15	0.975	0.907	0.798	0.509	0.184	0.125	0.000229	0	0
OF_MAG50	0.934	0.752	0.463	0.146	0.0312	0.0225	0.000171	0	0
OF_MAG88	0.977	0.911	0.807	0.526	0.195	0.132	0.000343	0	0
OF_MAG87	0.99	0.964	0.921	0.728	0.475	0.246	0.000457	0	0
OF_MAG85	0.904	0.636	0.249	0.0702	0.0144	0.0184	0.000114	0	0
OF_MAG84	0.994	0.978	0.952	0.832	0.683	0.405	0.0008	0	0
NOF_MAG27	0.829	0.359	0.0111	0.00439	0.0043	0.00794	0	0	0
NOF_MAG25	0.876	0.531	0.109	0.0488	0.0111	0.0167	5.72E-05	0	0
OF_MAG77	0.992	0.969	0.932	0.768	0.542	0.282	0.000514	0	0
OF_MAG78	0.979	0.919	0.825	0.552	0.216	0.148	0.000343	0	0
OF_MAG59	0.994	0.976	0.949	0.82	0.655	0.382	0.0008	0	0
OF_MAG56	0.876	0.531	0.109	0.0488	0.0111	0.0167	5.72E-05	0	0
OF_MAG72	0.979	0.919	0.825	0.552	0.216	0.148	0.000343	0	0
OF_MAG48	0.963	0.859	0.693	0.325	0.125	0.0682	0.000171	0	0
OF_MAG44	0.985	0.942	0.873	0.637	0.328	0.204	0.000343	0	0
NOF_MAG3	0.99	0.962	0.917	0.716	0.457	0.24	0.000457	0	0
NOF_MAG7	0.992	0.971	0.938	0.785	0.578	0.311	0.000514	0	0
NOF_MAG6	0.999	0.997	0.993	0.975	0.942	0.873	0.00269	0.000182	0

NOF_MAG9	0.993	0.972	0.94	0.792	0.593	0.325	0.000514	0	0
NOF_MAG10	0.988	0.954	0.9	0.682	0.405	0.227	0.0004	0	0
OF_MAG39	0.994	0.976	0.948	0.817	0.649	0.376	0.0008	0	0
OF_MAG36	0.99	0.963	0.919	0.724	0.469	0.244	0.000457	0	0
OF_MAG35	0.981	0.928	0.844	0.58	0.248	0.169	0.000343	0	0
OF_MAG34	0.973	0.897	0.777	0.468	0.166	0.113	0.000171	0	0
OF_MAG32	0.983	0.937	0.862	0.614	0.293	0.191	0.000343	0	0
OF_MAG31	0.841	0.401	0.0172	0.0121	0.00528	0.00864	0	0	0
OF_MAG30	0.986	0.946	0.883	0.66	0.365	0.218	0.0004	0	0
OF_MAG28	0.998	0.993	0.985	0.947	0.879	0.748	0.00252	0.000182	0
OF_MAG23	0.996	0.984	0.965	0.877	0.775	0.524	0.0016	0	0
OF_MAG22	0.995	0.98	0.957	0.848	0.718	0.441	0.00131	0	0
OF_MAG17	0.843	0.41	0.02	0.0154	0.00573	0.00938	0	0	0
OF_MAG10	0.982	0.933	0.854	0.599	0.271	0.18	0.000343	0	0
OF_MAG7	0.976	0.91	0.804	0.52	0.191	0.13	0.000286	0	0
OF_MAG2	0.846	0.419	0.0245	0.0197	0.00619	0.0102	5.72E-05	0	0
NOF_MAG31	0.975	0.906	0.796	0.503	0.181	0.123	0.000229	0	0
OF_MAG95	0.97	0.888	0.758	0.429	0.153	0.102	0.000171	0	0
OF_MAG94	0.916	0.684	0.332	0.0908	0.0169	0.0197	0.000171	0	0
OF_MAG93	0.852	0.442	0.0363	0.0284	0.00734	0.0121	5.72E-05	0	0
OF_MAG92	0.999	0.997	0.994	0.98	0.954	0.892	0.00269	0.000182	0
OF_MAG91	1	0.999	0.997	0.989	0.973	0.93	0.00509	0.000182	0
OF_MAG89	1	0.999	0.998	0.993	0.984	0.956	0.0477	0.00182	0.000206
OF_MAG70	0.997	0.988	0.974	0.909	0.831	0.641	0.002	0	0
OF_MAG64	0.91	0.662	0.292	0.0792	0.0155	0.0192	0.000171	0	0
OF_MAG65	0.847	0.423	0.0262	0.0213	0.00639	0.0105	5.72E-05	0	0

OF_MAG63	0.975	0.905	0.793	0.498	0.178	0.12	0.000171	0	0
NOF_MAG22	0.959	0.844	0.661	0.288	0.111	0.0607	0.000171	0	0
NOF_MAG20	0.995	0.98	0.957	0.849	0.72	0.444	0.00131	0	0
NOF_MAG19	0.965	0.868	0.713	0.35	0.132	0.0739	0.000171	0	0
NOF_MAG17	0.906	0.645	0.264	0.0729	0.0148	0.0187	0.000114	0	0
OF_MAG58	0.997	0.989	0.977	0.917	0.841	0.664	0.002	0	0
OF_MAG57	1	0.998	0.996	0.987	0.969	0.92	0.00292	0.000182	0
OF_MAG55	0.985	0.942	0.873	0.637	0.328	0.204	0.000343	0	0
OF_MAG54	1	0.999	0.997	0.99	0.976	0.935	0.0064	0.000182	0
OF_MAG53	0.998	0.993	0.985	0.948	0.88	0.75	0.00252	0.000182	0
OF_MAG51	0.996	0.987	0.971	0.899	0.812	0.599	0.00177	0	0
OF_MAG83	0.863	0.484	0.0663	0.0412	0.00973	0.0157	5.72E-05	0	0
OF_MAG81	0.969	0.882	0.744	0.402	0.145	0.0911	0.000171	0	0
NOF_MAG26	0.854	0.448	0.0398	0.0305	0.00769	0.0126	5.72E-05	0	0
OF_MAG76	0.967	0.876	0.729	0.375	0.138	0.0809	0.000171	0	0
OF_MAG75	0.999	0.996	0.992	0.97	0.93	0.852	0.00257	0.000182	0
OF_MAG74	0.974	0.9	0.784	0.48	0.17	0.116	0.000171	0	0
OF_MAG71	0.977	0.913	0.81	0.531	0.198	0.135	0.000343	0	0
OF_MAG73	0.924	0.712	0.384	0.107	0.0198	0.0205	0.000171	0	0
OF_MAG49	1	0.999	0.998	0.993	0.985	0.959	0.0827	0.0107	0.00103
NOF_MAG11	0.973	0.897	0.777	0.468	0.166	0.113	0.000171	0	0
OF_MAG43	1	0.999	0.998	0.994	0.985	0.959	0.0898	0.014	0.00124
OF_MAG42	0.999	0.995	0.99	0.963	0.914	0.82	0.00252	0.000182	0
NOF_MAG8	0.995	0.982	0.961	0.862	0.748	0.479	0.00149	0	0
NOF_MAG4	0.986	0.946	0.883	0.66	0.365	0.218	0.0004	0	0
NOF_MAG5	0.998	0.993	0.985	0.948	0.88	0.75	0.00252	0.000182	0

OF_MAG40	0.998	0.994	0.987	0.953	0.891	0.768	0.00252	0.000182	0
OF_MAG37	0.998	0.993	0.986	0.95	0.886	0.757	0.00252	0.000182	0
OF_MAG33	0.997	0.988	0.974	0.908	0.829	0.636	0.00194	0	0
OF_MAG29	0.998	0.993	0.985	0.949	0.883	0.754	0.00252	0.000182	0
OF_MAG27	0.996	0.987	0.971	0.899	0.812	0.599	0.00177	0	0
OF_MAG26	0.998	0.993	0.985	0.948	0.88	0.75	0.00252	0.000182	0
OF_MAG25	0.985	0.944	0.877	0.648	0.346	0.211	0.0004	0	0
OF_MAG24	0.998	0.994	0.987	0.955	0.895	0.775	0.00252	0.000182	0
OF_MAG21	0.996	0.985	0.968	0.887	0.791	0.556	0.00171	0	0
OF_MAG19	1	0.999	0.997	0.99	0.976	0.937	0.00857	0.000182	0
OF_MAG20	0.995	0.981	0.958	0.851	0.725	0.449	0.00131	0	0
OF_MAG18	0.991	0.966	0.926	0.746	0.503	0.258	0.000457	0	0
OF_MAG16	0.974	0.903	0.79	0.492	0.175	0.119	0.000171	0	0
OF_MAG14	0.997	0.987	0.972	0.9	0.814	0.605	0.00177	0	0
OF_MAG8	0.994	0.977	0.951	0.826	0.671	0.395	0.0008	0	0
OF_MAG6	0.995	0.983	0.962	0.867	0.757	0.493	0.00154	0	0
OF_MAG4	0.995	0.98	0.957	0.849	0.72	0.444	0.00131	0	0
NOF_MAG1	0.999	0.998	0.996	0.984	0.963	0.906	0.00269	0.000182	0
OF_MAG12	0.993	0.972	0.94	0.792	0.593	0.325	0.000514	0	0
OF_MAG47	0.993	0.973	0.942	0.799	0.61	0.341	0.000514	0	0

Supplementary Table 7. **Detailed information of elemental cycling functional genes.**

KO number	Gene ID	Description	Gene type
K00370	*narG*	nitrate reductase / nitrite oxidoreductase, alpha subunit [EC:1.7.5.1 1.7.99.-]	N-related gene
K00368	*nirK*	nitrite reductase (NO-forming) [EC:1.7.2.1]	N-related gene
K15864	*nirS*	nitrite reductase (NO-forming) / hydroxylamine reductase [EC:1.7.2.1 1.7.99.1]	N-related gene
K00376	*nosZ*	nitrous-oxide reductase [EC:1.7.2.4]	N-related gene
K01113	*phoD*	alkaline phosphatase D [EC:3.1.3.1]	P-related gene
K06137	*pqqC*	pyrroloquinoline-quinone synthase [EC:1.3.3.11]	P-related gene
K00394	*aprA*	adenylylsulfate reductase, subunit A [EC:1.8.99.2]	S-related gene
K00395	*aprB*	adenylylsulfate reductase, subunit B [EC:1.8.99.2]	S-related gene
K11181	*dsrB*	dissimilatory sulfite reductase beta subunit [EC:1.8.99.5]	S-related gene
K17222	*soxA*	L-cysteine S-thiosulfotransferase [EC:2.8.5.2]	S-related gene
K17224	*soxB*	S-sulfosulfanyl-L-cysteine sulfohydrolase [EC:3.1.6.20]	S-related gene
K17225	*soxC*	sulfane dehydrogenase subunit SoxC	S-related gene
K22622	*soxD*	S-disulfanyl-L-cysteine oxidoreductase SoxD [EC:1.8.2.6]	S-related gene
K17223	*soxX*	L-cysteine S-thiosulfotransferase [EC:2.8.5.2]	S-related gene
K17226	*soxY*	sulfur-oxidizing protein SoxY	S-related gene
K17227	*soxZ*	sulfur-oxidizing protein SoxZ	S-related gene
K07306	*dmsA*	anaerobic dimethyl sulfoxide reductase subunit A [EC:1.8.5.3]	S-related gene
K21310	*mddA*	methanethiol S-methyltransferase [EC:2.1.1.334]	S-related gene
K02588	*nifH*	nitrogenase iron protein NifH	N-related gene
K22357	*amoA*	alkene monooxygenase alpha subunit [EC:1.14.13.69]	N-related gene
KO4561	*norB*	nitric oxide reductase subunit B [EC:1.7.2.5]	N-related gene
K10944	*pmoA-amoA*	methane/ammonia monooxygenase subunit A [EC:1.14.18.3 1.14.99.39]	C-related gene
K00399	*mcrA*	methyl-coenzyme M reductase alpha subunit [EC:2.8.4.1]	C-related gene

K16157	*mmoX*	methane monooxygenase component A alpha chain [EC:1.14.13.25]	C-related gene
K11180	*dsrA*	dissimilatory sulfite reductase alpha subunit [EC:1.8.99.5]	S-related gene
K17486	*dmdA*	dimethylsulfoniopropionate demethylase [EC:2.1.1.269]	S-related gene

Supplementary Table 8. **Detailed information on variables in SEM.**

Latent Variable	Observable Variable	Units
Climate	mean annual temperature	°C
	annual precipitation	mm
Fertilizer type	Fertilizer type	
Soil	Total nitrogen	g/kg
	Organic carbon density	kg/m^3
	Soil organic carbon	g/kg
	pH	
Community diversity	Shannon index	
	Richness index	
Elements metabolism	Abundance of Carbon Metabolism	copies/cell
	Abundance of Nitrogen Metabolism	copies/cell
	Abundance of Phosphorus Metabolism	copies/cell
	Abundance of Sulfur Metabolism	copies/cell
	Abundance of Potassium Metabolism	copies/cell
ARG & MRG	ARG abundance	copies/cell
	MRG abundance	copies/cell
Co-existence of ARGs and MRGs	Abundance of ARG-MRG-carrying contigs	copies/cell
