# Supplementary material for: Organic fertilization co-selects genetically linked antibiotic and metal(loid) resistance genes in global soil microbiome
Source: Nat Commun. 2024 Jun 17;15:5168. doi: 10.1038/s41467-024-49165-5 (PMC11183072; doi:10.1038/s41467-024-49165-5)
Supplement: Supplementary file 3 — Reporting Summary [file 41467_2024_49165_MOESM3_ESM.pdf]

nature portfolio

Corresponding author(s): Si-Yu Zhang

Last updated by author(s): May 7, 2024

Reporting Summary

Nature Portfolio wishes to improve the reproducibility of the work that we publish. This form provides structure for consistency and transparency in reporting. For further information on Nature Portfolio policies, see our Editorial Policies and the Editorial Policy Checklist.

Statistics

For all statistical analyses, confirm that the following items are present in the figure legend, table legend, main text, or Methods section.

n/a	Confirmed	
☐	☒	The exact sample size (*n*) for each experimental group/condition, given as a discrete number and unit of measurement
☐	☒	A statement on whether measurements were taken from distinct samples or whether the same sample was measured repeatedly
☐	☒	The statistical test(s) used AND whether they are one- or two-sided<br>*Only common tests should be described solely by name; describe more complex techniques in the Methods section.*
☐	☒	A description of all covariates tested
☐	☒	A description of any assumptions or corrections, such as tests of normality and adjustment for multiple comparisons
☐	☒	A full description of the statistical parameters including central tendency (e.g. means) or other basic estimates (e.g. regression coefficient) AND variation (e.g. standard deviation) or associated estimates of uncertainty (e.g. confidence intervals)
☐	☒	For null hypothesis testing, the test statistic (e.g. *F*, *t*, *r*) with confidence intervals, effect sizes, degrees of freedom and *P* value noted<br>*Give P values as exact values whenever suitable.*
☒	☐	For Bayesian analysis, information on the choice of priors and Markov chain Monte Carlo settings
☒	☐	For hierarchical and complex designs, identification of the appropriate level for tests and full reporting of outcomes
☒	☐	Estimates of effect sizes (e.g. Cohen's *d*, Pearson's *r*), indicating how they were calculated

Our web collection on statistics for biologists contains articles on many of the points above.

Software and code

Policy information about availability of computer code

Data collection	The software used in this study includes kingfisher (wwood.github.io/kingfisher-download); fastp v0.22.0; MEGAHIT v1.2.9; MetaWRAP v1.2.1; CheckM v1.1.3; dRep v3.3.0; Prodigal v2.6.3; GTDB-Tk v2.1.0; tvBOT v2.5.0; BLAT v2.3.4.1; MicrobeCensus v1.1.0; Kraken2 v2.07; Bracken v2.7; DIAMOND v2.0.14.152; Gephi v0.9.2; PlasFlow; multitrim (https://github.com/KGerhardt/multitrim); SortMeRNA v4.3.4; R v4.3.1.
Data analysis	All the scripts and codes for classifying samples and machine learning used in this study were available online at Code Ocean (https://doi.org/10.24433/CO.8910377.v1).

For manuscripts utilizing custom algorithms or software that are central to the research but not yet described in published literature, software must be made available to editors and reviewers. We strongly encourage code deposition in a community repository (e.g. GitHub). See the Nature Portfolio guidelines for submitting code & software for further information.

Data

Policy information about availability of data

All manuscripts must include a data availability statement. This statement should provide the following information, where applicable:

- Accession codes, unique identifiers, or web links for publicly available datasets
- A description of any restrictions on data availability
- For clinical datasets or third party data, please ensure that the statement adheres to our policy

The accession codes for the 511 collected global soil metagenomes are available in Supplementary Table 1. The 12 metagenomic and metatranscriptomic raw sequencing data generated in this study have been deposited in the National Center for Biotechnology Information (NCBI) SRA database (https://

www.ncbi.nlm.nih.gov/sra) under the BioProject PRJNA1068274 and PRJNA1068685, respectively. Source data were provided with this paper.

Research involving human participants, their data, or biological material

Policy information about studies with human participants or human data. See also policy information about sex, gender (identity/presentation), and sexual orientation and race, ethnicity and racism.

Reporting on sex and gender	This item was not used in this study, which did not involve human participants, their data, or biological material.
Reporting on race, ethnicity, or other socially relevant groupings	This item was not used in this study, which did not involve human participants, their data, or biological material.
Population characteristics	This item was not used in this study, which did not involve human participants, their data, or biological material.
Recruitment	This item was not used in this study, which did not involve human participants, their data, or biological material.
Ethics oversight	This item was not used in this study, which did not involve human participants, their data, or biological material.

Note that full information on the approval of the study protocol must also be provided in the manuscript.

Field-specific reporting

Please select the one below that is the best fit for your research. If you are not sure, read the appropriate sections before making your selection.

☐ Life sciences ☐ Behavioural & social sciences ☒ Ecological, evolutionary & environmental sciences

For a reference copy of the document with all sections, see nature.com/documents/nr-reporting-summary-flat.pdf

Ecological, evolutionary & environmental sciences study design

All studies must disclose on these points even when the disclosure is negative.

Study description	Antibiotic resistance genes and metal(loid) resistance genes coexist in organic fertilized agroecosystems based on their correlations in abundance, yet evidence for the genetic-linkage of ARG-MRG co-selected by organic fertilization remains elusive. Here, We collected 511 global farmland soil metagenomes to explore the effects of organic fertilizer application on ARG and MRG co-selection. We further validated the co-existence and co-regulation of the ARG-MRG pairs in genetic elements under increased arsenic stress by applying analyses of 12 metagenomes and metatranscriptomes collected from agricultural soils in China.
Research sample	Metagenomic samples from worldwide agricultural soils were retrieved from the Sequence Read Archive (SRA, https://www.ncbi.nlm.nih.gov/sra) database, the European Nucleotide Archive (ENA, https://www.ebi.ac.uk/ena/browser/home), the DNA Data Bank of Japan (DDBJ, https://www.ddbj.nig.ac.jp), and the National Genomics Data Center (NGDC, https://ngdc.cncb.ac.cn/) by searching for the keyword 'agricultural soil' in October 2022. Detailed information of 511 soil metagenomes is provided in Supplementary Table 1.<br><br>In addition, 12 soil samples were collected from four different paddy fields distributed in four cities in China including Yuchangping (CS_YCP_1/2/3, 28.1711 N, 112.6664 E) in July 2022, Huaihua (HN_HH_1/2/3, 27.8782 N, 110.2414 E) in April 2023, Baiyun (CD_BY_1/2/3, 29.6639 N, 111.0504 E) July 2022, and Qujiang (ZJ_QJ_1/2/3, 29.0609 N, 118.9941 E) in April 2023. Three replicates from the same field for each site, were collected from the soil surface (0 to 20 cm) by ZTL and SYZ. The 12 agricultural soil metagenomes and metatranscriptomes generated in this study were used to validate the co-existence and co-regulation of the ARG-MRG pairs under arsenic stress.
Sampling strategy	The data obtained from online databases were subsequently refined using the following criteria: (1) Plant-associated samples, such as rhizosphere and rhizoplane soils, were excluded; (2) Only topsoil (depth < 20 cm) metagenomic samples were retained; (3) Only paired-end sequencing reads were generated by Illumina shotgun platforms were included; and (4) The data size (number of bp) of every metagenomic sample was greater than 1 Gb.<br><br>Finally, a total of 511 agricultural soil metagenome datasets were obtained from online database at our best after excluded those do not meet our sampling criteria, which were critical for us to determine the global distribution of co-existence ARG-MRG in agricultural soil microbiome.<br><br>In addition, 12 soil samples collected from agricultural land in China were previously investigated for metalloid contamination level, and collected according to the level of arsenic content in the soil. The 12 agricultural soil metagenomes and metatranscriptomes generated in this study were used to validate the co-existence and co-regulation of the ARG-MRG pairs under arsenic stress, which facilitated our research.
Data collection	ZTL downloaded the 511 metagenomic datasets from online databases, including SRA, ENA, DDBJ, and NGDC database, and saved them on our lab server for further analyses.<br><br>ZTL and SYZ sampled the 12 soil samples from four different paddy fields in China located in Yuchangping (CS_YCP_1/2/3), Huaihua (HN_HH_1/2/3), Baiyun (CD_BY_1/2/3), and Qujiang (ZJ_QJ_1/2/3) , and the site information was recorded by pen and paper at the time of sampling.

Timing and spatial scale	The metagenomic samples collected from online databases (n=511) were downloaded in October 2022. And the latitude and longitude of the collected samples are provided in Supplementary Table 1.<br><br>The 12 soil samples were collected from four different paddy fields distributed in four cities in China including Yuchangping (CS_YCP_1/2/3, 28.1711 N, 112.6664 E) in July 2022, Huaihua (HN_HH_1/2/3, 27.8782 N, 110.2414 E) in April 2023, Baiyun (CD_BY_1/2/3, 29.6639 N, 111.0504 E) July 2022, and Qujiang (ZJ_QJ_1/2/3, 29.0609 N, 118.9941 E) in April 2023. Three replicates from the same field for each site, were collected from the soil surface (0 to 20 cm) by ZTL and SYZ.
Data exclusions	No data were excluded in the analyses.
Reproducibility	No experiments were involved in this study.
Randomization	Based on the data uploaded or corresponding article information, 109 of the 511 global agricultural soil samples were classified as fertilized with organic fertilizer (OF), and 109 samples were classified as not fertilized with organic fertilizer (NOF). Due to insufficient information on fertilization types, 293 of the 511 agricultural soil samples lacked information on fertilization types. The trained random forest (RF) classification model was built based on the information of the 109 identified NOF samples and 109 OF samples by the R package "randomForest" (v 4.7-1.1), and was subsequently used to classify 293 agricultural soil samples as NOF or OF samples (detailed in the Supplementary Results). For the samples collected from agricultural lands in China, the 12 soil samples were divided into relatively high levels of arsenic stress and relatively low levels of arsenic stress according to arsenic concentration in the soil.
Blinding	Blinding was not used in this study, because it was not influenced by the observer.

Did the study involve field work? ☒ Yes ☐ No

Field work, collection and transport

Field conditions	The temperature range at the time of 12 samples collection was 30 to 37 degrees Celsius. The total carbon (TC) concentration ranged from 22.00 to 41.00 g kg-1, total nitrogen (TN) concentration ranged from 1.73 to 2.41 g kg-1, total phosphorus (TP) concentration ranged from 0.37 to 1.60 g kg-1, and total sulfate (TS) concentration ranged from 0.19 to 0.49 g kg-1.
Location	The 12 soil samples located in Yuchangping (28.1711 N, 112.6664 E), Huaihua (27.8782 N, 110.2414 E), Baiyun (29.6639 N, 111.0504 E), and Qujiang (29.0609 N, 118.9941 E).
Access & import/export	We drive to the paddy fields, which located in four different cities in China, and sampled the 12 soils. The samples were placed in sterile plastic bags and transported by SF-express to the laboratory in East China Normal University on ice under permits of national and local governments.
Disturbance	This study did not cause any environmental disturbance.

Reporting for specific materials, systems and methods

We require information from authors about some types of materials, experimental systems and methods used in many studies. Here, indicate whether each material, system or method listed is relevant to your study. If you are not sure if a list item applies to your research, read the appropriate section before selecting a response.

Materials & experimental systems

n/a	Involved in the study
☒	☐ Antibodies
☒	☐ Eukaryotic cell lines
☒	☐ Palaeontology and archaeology
☒	☐ Animals and other organisms
☒	☐ Clinical data
☒	☐ Dual use research of concern
☒	☐ Plants

Methods

n/a	Involved in the study
☒	☐ ChIP-seq
☒	☐ Flow cytometry
☒	☐ MRI-based neuroimaging

Plants

Seed stocks	This item did not apply to this study because it did not involve plant-related research.
Novel plant genotypes	This item did not apply to this study because it did not involve plant-related research.
Authentication	This item did not apply to this study because it did not involve plant-related research.
